# CD4^+^ T cells display a spectrum of recall dynamics during re-infection with malaria parasites

**DOI:** 10.1038/s41467-024-49879-6

**Published:** 2024-06-28

**Authors:** Hyun Jae Lee, Marcela L. Moreira, Shihan Li, Takahiro Asatsuma, Cameron G. Williams, Oliver P. Skinner, Saba Asad, Michael Bramhall, Zhe Jiang, Zihan Liu, Ashlyn S. Kerr, Jessica A. Engel, Megan S. F. Soon, Jasmin Straube, Irving Barrera, Evan Murray, Fei Chen, Jason Nideffer, Prasanna Jagannathan, Ashraful Haque

**Affiliations:** 1 Department of Microbiology and Immunology, University of Melbourne, located at The Peter Doherty Institute for Infection and Immunity, Parkville, VIC Australia; 2 QIMR Berghofer Medical Research Institute, Herston, Brisbane, QLD Australia; 3 University of Queensland, Brisbane, QLD Australia; 4 Broad Institute of Harvard and MIT, Cambridge, MA USA; 5 Department of Medicine, Stanford University, CA, USA; 6 Department of Microbiology and Immunology, Stanford University, CA, USA

**Keywords:** Malaria, Immunological memory, CD4-positive T cells, Gene regulation in immune cells, Next-generation sequencing

## Abstract

Children in malaria-endemic regions can experience repeated *Plasmodium* infections over short periods of time. Effects of re-infection on multiple co-existing CD4^+^ T cell subsets remain unresolved. Here, we examine antigen-experienced CD4^+^ T cells during re-infection in mice, using scRNA-seq/TCR-seq and spatial transcriptomics. TCR transgenic T_EM_ cells initiate rapid Th1/Tr1 recall responses prior to proliferating, while GC Tfh counterparts are refractory, with T_CM_/Tfh-like cells exhibiting modest non-proliferative responses. Th1-recall is a partial facsimile of primary Th1-responses, with no upregulated effector-associated genes being unique to recall. Polyclonal, TCR-diverse, CD4^+^ T cells exhibit similar recall dynamics, with individual clones giving rise to multiple effectors including highly proliferative Th1/Tr1 cells, as well as GC Tfh and Tfh-like cells lacking proliferative capacity. Thus, we show substantial diversity in recall responses mounted by multiple co-existing CD4^+^ T cell subsets in the spleen, and present graphical user interfaces for studying gene expression dynamics and clonal relationships during re-infection.

## Abstract (web summary)

CD4^+^ T cells are known to be important in Plasmodium infection. Here the authors use mouse models to track antigen-experienced TCR transgenic and polyclonal CD4^+^ T cells during Plasmodium re-infection, and show different T cell phenotypes and varied responses in different areas of the spleen.

## Introduction

Malaria is caused by infection with *Plasmodium* parasites, with 249 million cases and 608,000 deaths in 2022^1^. It remains a major global health burden, with malaria morbidity and mortality highest amongst young children^1^. In highly endemic regions, children can experience repeated infections over short timeframes of weeks to months^2,3^. Although these cumulative infections eventually result in protection from severe and fatal malaria syndromes^4,5^, the impact of each new infection on ongoing cellular and humoral immune responses remains undefined.

CD4^+^ T cells orchestrate naturally-acquired immunity to blood-stage *Plasmodium* parasites. Since IgG from affinity-matured, class-switched B cells limit parasitemia, germinal centre (GC) T follicular helper (Tfh) cells are strongly implicated in blood-stage immunity^6,7^. T helper 1 (Th1) cells can also control blood-stage parasite numbers^8^, it is thought via the action of pro-inflammatory cytokines on phagocytes, while IL-10-producing type 1 regulatory T (Tr1) cells can protect against immune-pathology^9,10^. Hence immunity to blood-stage malaria likely depends on multiple effector and memory CD4^+^ T cell subsets. However, the impact of re-infection on these various cell states remains unclear. Assessment of peripheral blood CD4^+^ T cells in those living in highly endemic regions revealed an increased propensity for interleukin-10 production with increased age and exposure^11,12^. This suggests repeated infection alters the memory of CD4^+^ T cells qualitatively, although effects on multiple states, including GC Tfh and Tfh-like cells remain largely undefined. Studies of experimental vaccination indicate GC Tfh cells cease proliferation during primary responses, perhaps to maintain stringent B cell selection and to prevent autoimmunity^13,14^. The effect of rechallenge on pre-existing GC Tfh responses remains unclear, yet is relevant in cases of re-infection or “break-through” infection soon after primary infection or vaccination. It has been reported in murine models of viral infection and vaccination, that Tfh-like cells can proliferate and exhibit functional plasticity when adoptively transferred into naïve hosts^15–17^. Whether this occurs during re-infection with parasitic organisms remains unclear, although Latham et al. noted while Th1-like cells increased in number during re-infection with *Plasmodium*, Tfh-like cells did not, and GC Tfh cells contracted slightly over time^18^. These data suggest *Plasmodium-*specific CD4^+^ T cells exhibit heterogeneous responses during re-infection, although this remains to be fully explored.

More generally, our understanding of CD4^+^ T cell memory recall in experimental or clinical settings is largely focussed on T effector memory (T_EM_) cells, and their expression of a handful of molecules including cytokines IFN-γ, TNF, IL2, IL10, costimulatory molecules such as OX40, and proliferative markers, e.g. Ki67 and BrdU incorporation. The broader relationship between primary and recall CD4^+^ effector T cell responses is poorly defined.

Previously, we employed TCR-transgenic (PbTII) CD4^+^ T cells, specific for an epitope from *Plasmodium* Heat Shock Protein 90^19,20^, to map the transcriptional dynamics that accompany clonal expansion, Th1/Tfh effector fate choice^21,22^, and transit to memory or exhausted states in experimental primary malaria^23^. These studies suggested roles for inflammatory monocytes and B cells, and various genes in controlling Th1/Tfh fate choice, as well as revealing that memory emerges gradually over a 3–4 week period from effector counterparts. Importantly, droplet-based scRNA-seq at late timepoints suggested a complex landscape of cellular states amongst splenic PbTII cells comprised of Th1 T_EM_ cells, T central memory (T_CM_) cells, GC Tfh cells, Tfh cells, and other mixed phenotypes. Whether and how these transcriptionally diverse cells might respond during re-infection remained untested. Moreover, the relevance of TCR transgenic cells to highly diverse polyclonal responses was unclear.

Here, we map over time the in vivo responses of splenic antigen-experienced, TCR-transgenic and polyclonal CD4^+^ T cells during re-infection with *Plasmodium* parasites, using a combination of droplet-based scRNA-seq, VDJ-seq, spatial transcriptomics and computational modelling. We define CD4^+^ T cell heterogeneity before and after re-infection. We reveal differential recall dynamics, predict further biological processes and upregulated genes associated with re-infection with blood-stage parasites, and present these datasets in the following graphical user interface: https://haquelab.mdhs.unimelb.edu.au/cd4_re-infection.

## Results

### *Plasmodium*-specific PbTII cells exhibit a spectrum of transcriptional states in the spleen after primary infection and drug treatment

**Fig. 1:**
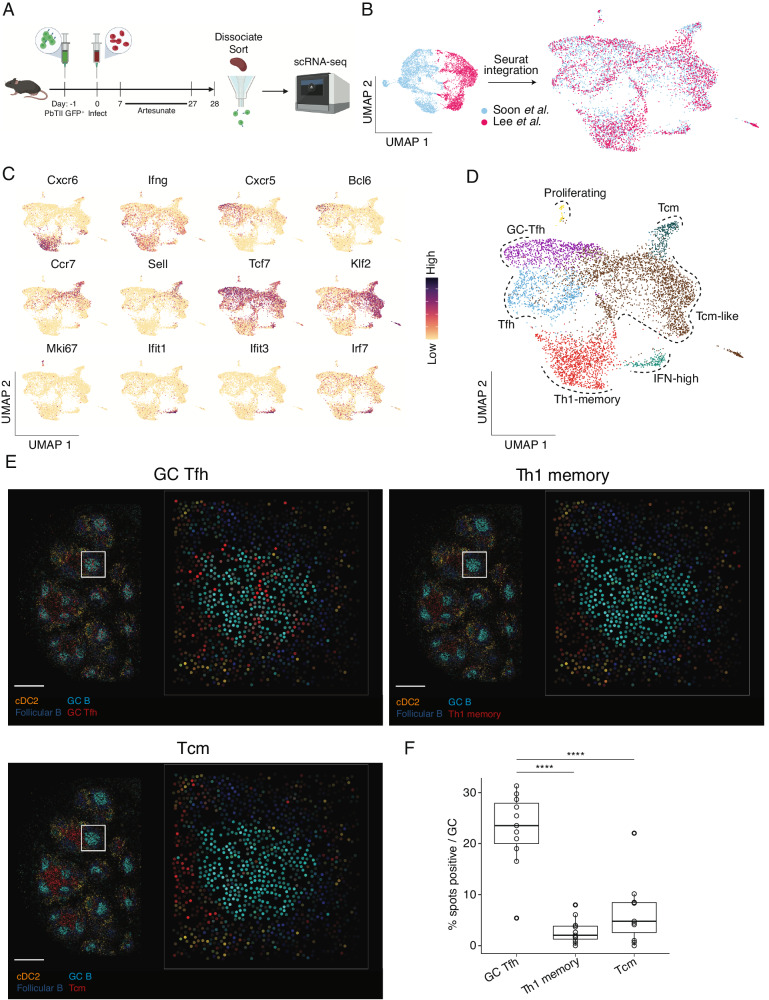
PbTII cells exhibit a spectrum of transcriptional states in the spleen after primary infection and antimalarial drug treatment. **A** Schematic of scRNA-seq experiment to study PbTII cells at day 28 post-infection. **B** UMAPs of PbTII cell-derived scRNA-seq data before and after integration of our two independent experiments, this study and Soon et al.^23^—cells coloured by experimental origin. **C** UMAPs of PbTII cell gene expression patterns for Th1 (*Cxcr6*, *Ifng*), Tfh (*Cxcr5*, *Bcl6*), T_CM_ (*Ccr7*, *Sell*, *Tcf7*, *Klf2*), proliferation (*Mki67*), and IFN-high (*Ifit1*, *Ifit3*, *Irf7*) genes. **D** UMAP of PbTII cell states depicted by colours and dotted boundaries. **E** Estimated cell-type abundance for GC Tfh, T_CM_, and Th1-memory (red), compared with the same cDC2, GC B cells and follicular B cells in each panel, assessed across a *Slide-seqV2* spatial transcriptomic map of mouse spleen at day 30 p.i.—scale bar: 500 µm: white boxes shown at higher magnification within each inset panel, with each dot representing a 10 µm bead. **F** Box plots showing % positive occurrence of GC Tfh, T_CM_, or Th1-memory cells in each GC (each open circle denoting 1 GC). Cell abundance (computed using *cell2location*) >0.2 is considered a positive occurrence. Centre line indicates median and box limits upper and lower quartiles; two-sided Wilcoxon signed rank test: ****P < 0.0002 (exact values: *P* = 0.00019 for GC Tfh vs. Tcm; *P* = 1.134 × 10^−5^ for GC Tfh vs. Th1-memory).

To map CD4^+^ T cell responses during re-infection of mice with malaria parasites, we first tested whether PbTIIs consistently seeded the spleen with heterogeneous antigen-experienced CD4^+^ T cells, after *Plasmodium chabaudi chabaudi* AS (*Pc*AS) infection and antimalarial treatment^23^. Splenic PbTIIs recovered 4 weeks after primary infection and treatment (Supplementary Fig. 1A), were processed by droplet-based scRNA-seq (Fig. 1A). After removal of low-quality transcriptomes and computational integration with our previous dataset (Fig. 1B; Supplementary Fig. 1B; Supplementary Fig. 1C), principal component analysis (PCA) followed by uniform manifold approximation and projection (UMAP) revealed similar proportions of cells from the two independent experiments in each transcriptionally defined cluster (Supplementary Fig. 1D), also observed with a second integration approach, *Harmony*^24^ (Supplementary Fig. 1E). This suggested PbTIIs adopted a consistent heterogeneous set of transcriptional states after primary infection and drug cure. Assessment of canonical naïve/Tcm (*Ccr7*, *Sell*, *Tcf7*, and *Klf2*), helper (*Cxcr6*, *Ifng*, *Cxcr5*, and *Bcl6*), proliferative (*Mki67*), and Type I IFN-associated (*Ifit1*, *Ifit3*, and *Irf7*) genes (Fig. 1C), supported the existence of GC Tfh (marked by high expression of *Pdcd1, Cxcr5* and *Bcl6*), Tfh, Th1-T_EM_, T_CM_, IFN-associated (IFN-high) and proliferating states (Fig. 1D). Some of these states appeared not as distinct clusters, but as a continuum (Fig. 1D).

To determine the location of cells transcriptionally annotated as GC Tfh, we conducted spatial transcriptomic assessment using *Slide-seqV2*, a technique offering near single-cell resolution^25,26^. As expected, discrete GC B cell transcriptomic regions were noted within B cell follicles in infected mice (Fig. 1E; Supplementary Fig. 2). GC Tfh transcriptomes derived from PbTII data, were enriched in GC regions compared to either Th1 or T_CM_ transcriptomes, which instead were confined to areas between B cell follicles, inferred to be T-cell zones enriched with cDC2 cells (Fig. 1F). This confirmed GC Tfh transcriptomes co-localised with GC B cells, with circulating memory states residing in T-cell zones. Thus, a variety of CD4^+^ T cell memory and Tfh states co-existed in distinct areas of the spleen after primary *Plasmodium* infection and anti-malarial drug treatment.

### Antigen-experienced CD4^+^ T cells display a variety of recall dynamics during re-infection

**Fig. 2:**
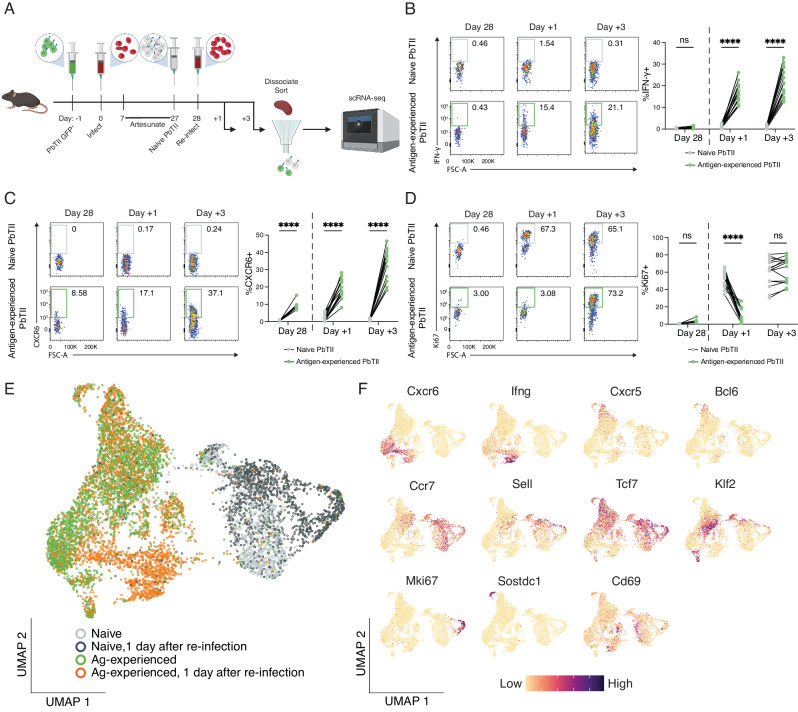
Re-infection triggers an early transcriptional response from Th1-memory PbTIIs. **A** Schematic of scRNA-seq experiment to study in vivo responses of antigen-experienced versus naïve PbTII cells during re-infection. **B–D** Representative FACS plots showing **B** IFN-γ, **C** CXCR6, and **D** Ki67 expression in antigen-experienced PbTII cells at day 28 p.i. and 1 and 3 days post re-infection, compared to co-transferred naïve comparator PbTIIs. Data combined from two independent experiments showing similar results (*n* = 9 mice for day 28 p.i, 15 mice each for 1 and 3 days post-re-infection); two-way ANOVA with Šídák’s multiple comparison testing; ****P < 0.0001. **E** UMAP of antigen-experienced PbTII cells and naïve comparators, prior to and 1 day after re-infection. **F** UMAPs of PbTII expression of various genes associated with Th1 (*Cxcr6*, *Ifng*), Tfh (*Cxcr5*, *Bcl6*), T_CM_ (*Ccr7*, *Sell*, *Tcf7*, *Klf2*), proliferation (*Mki67*), *Sostdc1*^+^ cells, and early activation (*Cd69*).

To map responses of heterogeneous splenic CD4^+^ T cells during homologous re-infection, we first determined by flow cytometry whether antigen-experienced PbTIIs could mount a pronounced recall response. Our previous study indicated a minority (~20–30%) of PbTIIs secreted IFN-γ without proliferating within 24 h^23^. Here, we extended these findings, confirming that in contrast to CTV-labelled naïve PbTIIs (Supplementary Fig. 3) co-transferred one day prior to re-infection (Fig. 2A), 10–30% of antigen-experienced PbTIIs (Supplementary Fig. 3) rapidly secreted IFN-γ and upregulated CXCR6 by day 1 to day 3 of re-infection (Fig. 2B and C), consistent with a pronounced Th1-recall response. In addition, the majority of PbTIIs, but not all, had upregulated Ki67 expression by day 3, suggesting a heterogeneous capacity for proliferation (Fig. 2D). Together, these data indicated that proliferative and Th1-recall responses were mounted by some but not all antigen-experienced PbTIIs.

To test for heterogeneity in recall, we examined antigen-experienced PbTIIs and naïve comparators by scRNA-seq 24 h after re-infection (Fig. 2E). Antigen-experienced cells enriched for Th1-markers *Cxcr6* and *Ifng* had indeed undergone transcriptomic change, suggestive of rapid Th1-recall (Fig. 2F). In stark contrast, antigen-experienced PbTIIs enriched for *Tcf7* and either Tfh markers *Bcl6* and *Cxcr5*, or T_CM_ markers, *Sell, Ccr7* and *Klf2*, exhibited no transcriptomic change (Fig. 2F). We also noted a minor population of GC Tfh-like cells that expressed high levels of *Sostdc1* (Fig. 2F), previously implicated in promoting T follicular regulatory cells^27^. Finally, consistent with flow cytometric assessment, only naïve PbTII control cells upregulated *Mki67* over this timeframe (Fig. 2F). Therefore, during the first 24 h of re-infection, Th1-memory PbTIIs mounted a rapid non-proliferative effector recall response, while GC Tfh, Tfh, T_CM_ and *Sostdc1*^*+*^ GC Tfh states remained transcriptionally unaltered. These data revealed striking heterogeneity in the early recall response of splenic, antigen-experienced CD4^+^ T cells of the same peptide-specificity.

**Fig. 3:**
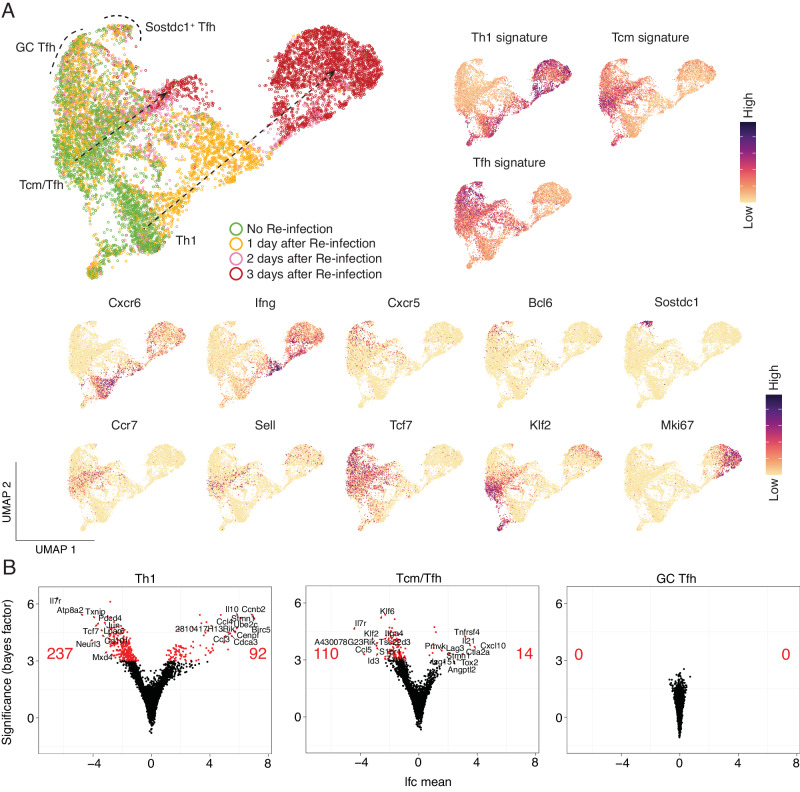
PbTII cells exhibit a spectrum of recall dynamics during re-infection. **A** (*Left*) UMAP representation of antigen-experienced PbTII cells prior to and 1, 2, and 3 days post-re-infection; two apparent trajectories indicated by arrows; GC Tfh cells and *Sostdc1*^+^ Tfh cells marked with dotted boundaries. (*Right*) Th1, Tcm, and Tfh signature scores. (*Bottom*) Expression patterns for various genes associated with Th1 (*Cxcr6*, *Ifng*), Tfh (*Cxcr5*, *Bcl6*), *Sostdc1*^+^ cells, T_CM_ (*Ccr7*, *Sell*, *Tcf7*, *Klf2*), and proliferation (*Mki67*) states. **B** Volcano plots depicting the number and LogFC (lfc mean) of differentially expressed genes (genes with Bayes factor > 3) from comparing Th1 cells (Left), T_CM_/Tfh cells (Right), and GC Tfh cells (Bottom) prior to and post re-infection. Number of significantly upregulated/downregulated genes and the top 10 upregulated/downregulated genes annotated on volcano plots.

To further test the apparent refractory nature of splenic Tfh and T_CM_ states relative to Th1-memory during re-infection, we extended our scRNA-seq analysis of antigen-experienced PbTIIs from day 1 to days 2 and 3 after re-infection, a time period during which flow cytometry had suggested robust Th1-recall and the emergence of a proliferative state (Fig. 2B–D). Low-dimensional UMAP embeddings after PCA, and annotation according to timepoint suggested three main trajectories, based on gene expression signatures and individual canonical genes, corresponding to Th1, T_CM_, and Tfh-like states (Fig. 3A). Together, these suggested that Th1-recall dynamics were characterised by rapid immune effector expression followed by cellular proliferation by day 3. In contrast, GC Tfh and *Sostdc1*^*+*^ GC Tfh appeared unaltered over the three-day period. T_CM_/Tfh-like cells displayed intermediate transcriptional change by day 3, notably devoid of *Mki67* upregulation, suggesting this cell state had not initiated proliferation (Fig. 3A). Differential gene expression analysis for each inferred cell state, prior to versus the apparent peak of their recall response suggested that gene downregulation was a feature for Th1-like T_EM_ cells, including, as expected, *Il7r* and *Tcf7* amongst the 237 downregulated genes (Fig. 3B). Th1-like T_EM_ cells upregulated 92 genes including expected secreted effectors *Ifng, Il10, Ccl4, and Ccl3*, as well as ~30 cell cycle genes including *Ccnb2*, *Cdca3* and *Cenpf*, consistent with some temporal overlap of proliferation with rapid effector function. T_CM_/Tfh-like cells downregulated 110 genes and upregulated only 14 genes, including *Tnfrsf4, Il21, Cxcl10* and *Lag3*, while GC Tfh cells exhibited no differentially expressed genes (Fig. 3B; Supplementary Data 1–3). Together, these data suggested GC Tfh cells were entirely refractory to transcriptional change over the first 3 days of re-infection, T_CM_/Tfh-like cells exhibited a modest non-proliferative response, while Th1-memory cells mounted a rapid, dynamic response marked by early *Ifng* and *Il10* expression and subsequent proliferation.

### Th1-recall features two waves of RNA processing associated with rapid effector function and subsequent proliferation

**Fig. 4:**
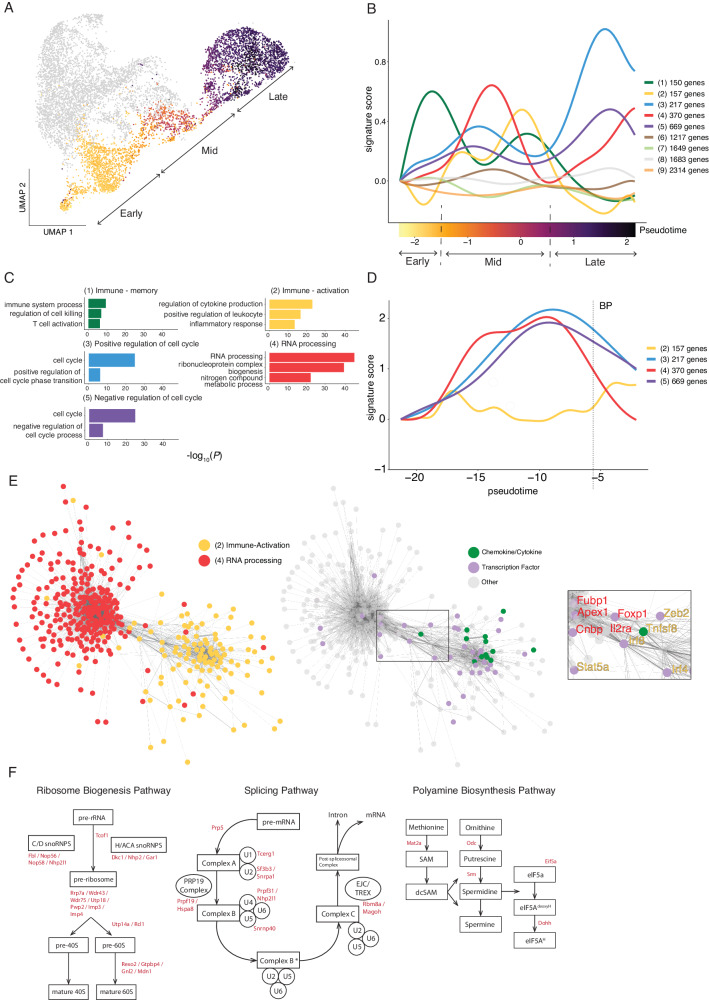
Transcriptome dynamics of Th1-recall predicts suggest early RNA processing associated with rapid effector function and subsequent proliferation. **A** UMAP of antigen-experienced PbTII cells prior to and 1, 2, and 3 days post-re-infection, with only Th1-like cells coloured according to inferred pseudotime values (from 1-dimensional BGPLVM, *GPfates*), split into early, mid and late-pseudotime. **B** Expression dynamics for pseudo-temporally variable genes in Th1-like cells along pseudotime, genes grouped according to similar dynamics, represented as signature scores. **C** Summary of gene ontology enrichment analysis of biological processes associated with genes for selected groups from (**B**). *X*-axis represents negative log-transformed *P*-values that indicate the extent of enrichment of biological processes; Fisher’s exact test is used to identify over-represented GO terms. **D** Expression dynamics for gene groups 2–5 for Th1-like PbTII cells during primary infection^23^; BP denotes Th1/Tfh bifurcation point. **E** Co-expression network analysis of genes (represented as nodes) in Dynamics 2 and 4. Edge weight corresponds to Spearman’s rho values (only showing rho > 0.2). Gene labels in the inset coloured according to the dynamic of origin. **F** Schematics of enriched biological pathways associated with genes from Dynamic 4. Genes found in Dynamic 4 are highlighted in red.

Since flow cytometric and scRNA-seq assessment of PbTIIs during re-infection had revealed temporally distinct periods for upregulation of IFN-γ compared to Ki67, we reasoned that a coordinated series of transcriptional changes accompanied the progression of Th1-memory cells from a quiescent state prior to re-infection, to effector and proliferative states during re-infection. To map Th1-recall dynamics, we segregated those PbTII recall transcriptomes with prominent Th1 signatures (Table 1 and Fig. 3A), and performed pseudo-temporal ordering via Bayesian Gaussian Processes Latent Variable Modelling (BGPLVM) using *GPfates*^22^. This generated a progression of Th1 transcriptomes that correlated with sampled time points (Fig. 4A). We then grouped all highly dynamic genes according to their expression dynamics using *spatialDE*^28^ (Supplementary Data 4). This revealed 9 distinct dynamics, ranging in size from 150 genes to 2314 genes (Fig. 4B). Gene Ontology (GO) enrichment analysis indicated some immune-associated genes in Dynamic 1, e.g. *Tcf7, Il7r*, and *Bcl2* rapidly waned after activation (Fig. 4B and C). These were followed by four prominent waves of transcription, with Dynamics 3 and 5 emerging last, comprised of 886 genes associated with cellular proliferation (Fig. 4B and C) and preceded by “immune-activation” genes including *Ifng*, *Tnf*, *Il2*, and *Tbx21* in Dynamic 2. In addition, Dynamic 4 appeared bi-phasic, with 370 genes associated with mRNA processing (Fig. 4C). These appeared upregulated earlier than cellular proliferation genes, yet had largely overlapped with them during primary infection (Fig. 4D). In addition, RNA processing genes appeared to overlap in their dynamics with immune activation genes, raising the hypothesis that genes within these groupings might be co-transcriptionally regulated. Transcriptional network analysis of Dynamics 2 and 4, specifically during mid-pseudotime, revealed a possible linking of the two (Fig. 4E), with several transcription factors implicated (*Irf8, Irf4, Foxp1, Zeb2, Stat5a, Apex1, Cnbp* and *Fubp1*), as well as *Il2ra* and *Tnfsf8* (encoding CD30L) (Fig. 4E). However, this apparent link was not observed at later points in pseudotime (Supplementary Fig. 4A). Instead, genes associated with cellular proliferation (Dynamics 3 and 5), were transcriptionally correlated with RNA processing (Dynamic 4) at both mid- and late pseudotime (Supplementary Fig. 4B), suggesting a possible functional link between these transcriptional networks which lasted longer than with immune activation genes (Dynamic 2). Further examination of genes within Dynamic 4 identified three RNA-associated pathways: ribosome biogenesis^29^ and splicing pathways^30^, which are involved in post-transcriptional/translational processes, and in addition, polyamine metabolism, previously reported to influence CD4^+^ T cell fate during primary immune responses^31^ (Fig. 4F). Together our data highlight that in contrast to primary Th1 responses, Th1-recall is characterised by early transcription of genes required for RNA processing, which is further amplified as cells progress towards proliferation, after a burst of rapid immune function. Furthermore, our data suggest that during recall, genes controlling cellular proliferation are transcriptionally unlinked to those mediating rapid effector function.

**Table 1:** Gene lists employed to discriminate CD4^+^ T cell states

	Genes
Th1	*S100a4, Cxcr6, Nkg7, Gzmb, Ccr5, Lgals3*
T_CM_	*Sell, Ccr7, Tcf7, Klf2*
Tfh	*Cxcr5, Tox2, Asap1, Tnfsf8, Slamf6, Tbc1d4*

### Th1-recall is characterised by upregulation of a select few effector genes

**Fig. 5:**
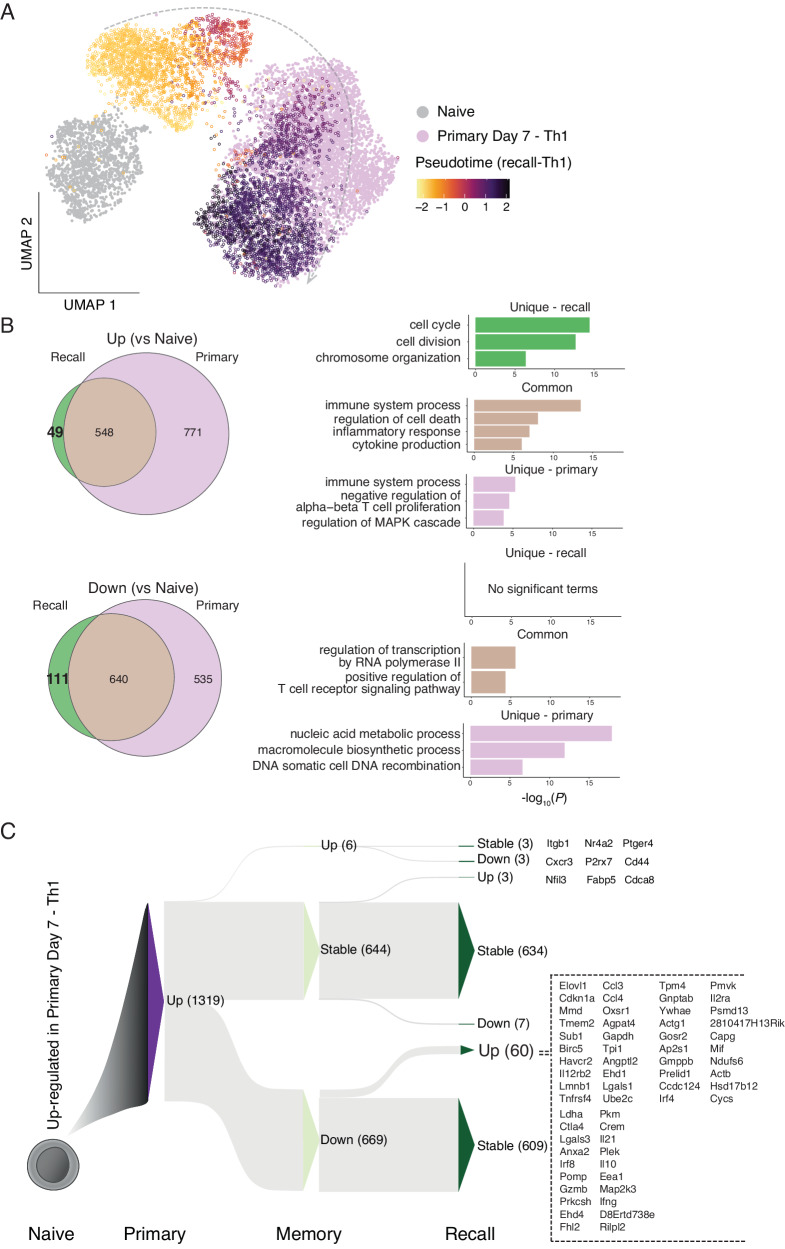
Th1-recall is characterised by the upregulation of a select few genes. **A** UMAP of PbTII cells after *scVI* integration of naïve and primary Th1 scRNA-seq data from Soon et al.^23^. with Th1-recall scRNA-seq in this study (open circles) coloured according to pseudotime values (after 1-dimensional BGPLVM)—apparent Th1-recall trajectory indicated with an arrow. **B** (Left) Venn diagrams showing overlap in lists of differentially expressed genes (Bayes factor > 3) from peak Th1-primary (relative to naïve PbTIIs), compared to peak Th1-recall (relative to naïve PbTIIs). (Right) Summary of gene ontology enrichment analysis of biological processes associated with genes common or unique to each comparison; Fisher’s exact test used to identify over-represented GO terms. **C** Sankey plot schematic summarising dynamics of 1319 genes initially upregulated in Th1-primary cells (compared to naïve PbTIIs), as cells proceed to memory and then to peak Th1-recall (Up: upregulated, Down: downregulated, Stable: no significant change; at each stage in reference to the preceding stage). Full list of all 60 genes upregulated from Th1-memory to recall is shown on the right.

We next searched for genes uniquely expressed during Th1-recall compared to primary Th1 responses. We computationally integrated Th1-recall transcriptomes across pseudotime with those from naïve and primary Th1 PbTIIs from our previous study^23^, using *single-cell Variational Inference* (*scVI*) to account for batch effects^32^. UMAP visualisation suggested naïve cells and Th1-memory cells prior to re-infection were transcriptionally distinct from each other and from primary Th1 cells (Fig. 5A). Importantly, Th1-recall transcriptomes at later stages of recall largely overlapped with primary Th1, suggesting a substantial similarity between the two, particularly at the peak of Th1 activity. This was corroborated by a relative lack (49 genes) of uniquely upregulated genes in Th1-recall compared to primary Th1 (both compared to naïve PbTIIs) (Fig. 5B). GO term enrichment analysis revealed the 49 uniquely upregulated genes were associated with cell cycle (Fig. 5B). Instead, Th1-recall upregulated genes (compared to naïve) were essentially a smaller subset (548 out of 1319) of those upregulated in primary Th1 (Fig. 5B). Together, our data support the view that no unique genes marked Th1-recall over primary Th1 responses, and moreover that Th1-recall represented a trimmed down version of primary responses.

Finally, we consolidated in a single map the transcriptional path as primary Th1 cells transitioned to Th1-memory and recall (1319 upregulated genes in primary Th1 cells shown in Fig. 5C and 1175 downregulated genes in primary Th1 cells shown in Supplementary Fig. 5). Out of 1319 upregulated genes, ~50% reverted to naïve levels in Th1-memory, leaving 634 genes stably upregulated in memory and recall (Fig. 5C; Supplementary Data 5). While immunological roles for many of these genes, including *Cxcr6* and *Tbx21* are well established, at least during primary responses, others, such as *Maf* remained to be studied in memory CD4^+^ T cells in vivo. Crucially, during Th1-recall only 4.8% (63/1319) of genes were further upregulated compared to quiescent Th1-memory cells (Fig. 5C). These included secreted molecules, *e.g. Ifng, Il10, Il21, Ccl3, Ccl4* and *Gzmb*, costimulatory markers or receptors (*Havcr2, Ctla4, Il2ra, Il12rb2, Tnfrsf4*) and transcription factors *Irf4* and *Irf8* (Fig. 5C). Taken together, these data indicate that Th1-recall is not characterised by transcriptional upregulation of hundreds or thousands of genes as observed during primary infection, but instead of a small gene set highly enriched in chemokine-, cytokine-, and co-stimulation-associated markers.

### TCR diverse, antigen-experienced CD4^+^ T cells in the spleen also exhibit varied propensity for recall during re-infection

**Fig. 6:**
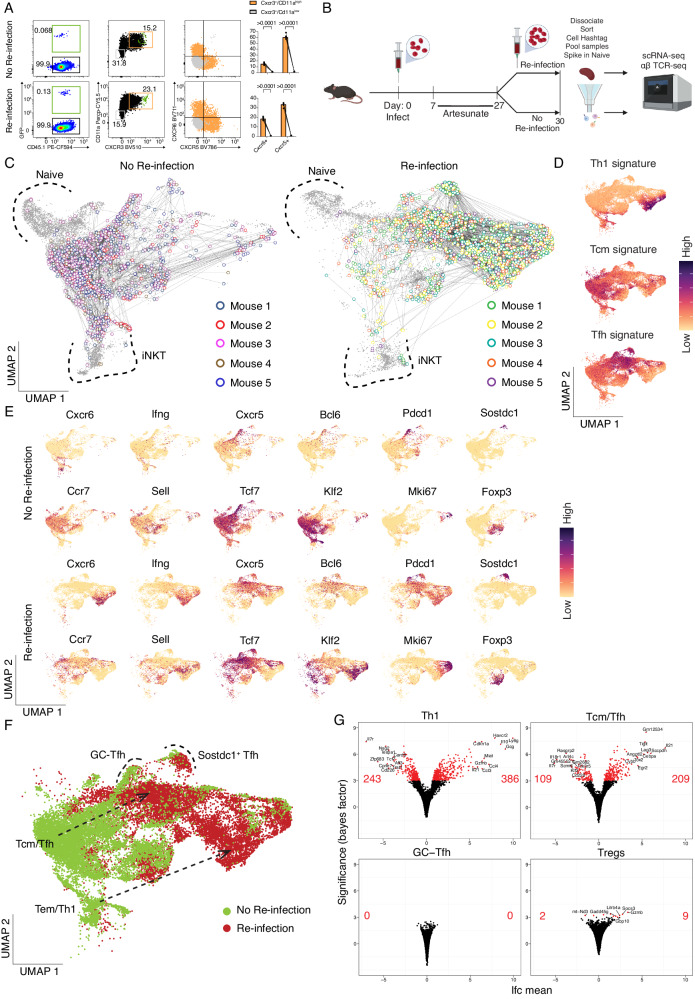
TCR-diverse, antigen-experienced CD4^+^ T cells exhibit varied recall responses during re-infection. **A** Representative FACS plot showing GFP^+^ PbTII cells, CD11a and CXCR3 expression, and CXCR6 and CXCR5 expression (orange: gated on CD11a^hi^CXCR3^+^, grey: gated on CD11a^lo^CXCR3^−^) in CD4^+^ T cells prior to and 3 days after re-infection. Graphs show the percentage of CXCR6^+^ and CXCR5^+^ cells from CD11a^hi^CXCR3^+^ versus CD11a^lo^CXCR3^−^ CD4^+^ T cells (*n* = 5 mice). Statistical analysis was performed using a Wilcoxon signed-ranks test. ****P < 0.0001. **B** Schematic of scRNA-seq and TCR-seq experiment to study CD11a^hi^CXCR3^+^ polyclonal CD4^+^ T cells prior to and 3 days after re-infection. **C** UMAP of CD11a^hi^CXCR3^+^ and inferred naïve (CD11a^lo^CXCR3^-^) polyclonal CD4^+^ T cells. Cells sharing the same TCR chains connected with edges. Only families with four or more cells sharing the same TCR chains are shown in the plot. Naïve cells and iNKT cells are marked with dotted boundaries. **D** UMAP of CD11a^hi^CXCR3^+^ cells expressing Th1, Tcm, and Tfh signature scores. **E** UMAP of CD11a^hi^CXCR3^+^ cells expressing genes associated with Th1 (*Cxcr6*, *Ifng*), Tfh (*Cxcr5*, *Bcl6*, *Pdcd1*), T_CM_ (*Ccr7*, *Sell*, *Tcf7*, *Klf2*), proliferation (*Mki67*), *Sostdc1*^+^ cells, and Treg (*Foxp3*) cells. **F** UMAP of CD11a^hi^CXCR3^+^ polyclonal CD4^+^ T cells prior to and 3 days post-re-infection. Differentiation trajectories are indicated with arrows. GC Tfh cells and *Sostdc1*^+^ Tfh cells are marked with dotted boundaries. **G** Volcano plots showing the number of differentially expressed genes (genes with Bayes factor > 3) comparing prior to and after re-infection for each inferred state: Th1 cells (Top-Left), T_CM_/Tfh cells (Top-Right), GC Tfh cells (Bottom-Left), and Treg cells (Bottom-Right). Number of significantly upregulated/downregulated genes and the top 10 upregulated/downregulated genes annotated on volcano plots.

To determine the relevance of our findings to TCR diverse polyclonal CD4^+^ T cells, we first determined prior to and after *PcAS* re-infection if co-expression of the markers CD11a, as previously reported^33–35^, and CXCR3 (as suggested from our previous studies^23,36^) would permit flow-cytometric enrichment of polyclonal, activated CD4^+^ T cells (Fig. 6A), with PbTIIs also examined as comparators. Firstly, PbTIIs prior to and 2 days after re-infection expressed high levels of CD11a and CXCR3 (Fig. 6A). Similarly, CD11a^lo^ CXCR3^lo^ polyclonal cells exhibited no upregulation of canonical Tfh/Th1 markers CXCR5 or CXCR6, while comparator CD11a^hi^ CXCR3^hi^ cells expressed high levels of both (Fig. 6A). This suggested that flow-cytometric sorting based on CD11a/CXCR3 would allow for enrichment of *Plasmodium-*specific antigen-experienced CD4^+^ T cells.

Next, we performed droplet-based scRNA-seq and VDJ sequencing on CD11a^hi^ CXCR3^hi^ polyclonal CD4^+^ T cells recovered from the spleens of individual mice prior to, and 3 days after re-infection, with a 5% spike-in of CD11a^lo^ CXCR3^lo^ control cells (Fig. 6B). After de-multiplexing, removal of doublets and low-quality transcriptomes (Supplementary Fig. 6A), TCR genes were removed from the gene expression matrix (Supplementary Fig. 6B) and contaminating non-CD4^+^ T cells were removed (Supplementary Fig. 6C). We annotated naïve cells based on frequency, lack of clonal connections (Fig. 6C), and low expression of *Cxcr3* (Supplementary Fig. 6D), as well as noting a minor contaminant of invariant Natural Killer T cells (iNKT), based on expression of semi-invariant TCR chains and *Zbtb16* (Supplementary Fig. 6D)^37^. UMAP visualisation prior to and 3 days after re-infection revealed heterogeneous populations of polyclonal CD4^+^ T cells (Fig. 6C) with substantial sharing of TCR chains within and across certain transcriptomically distinct clusters in every mouse (Fig. 6C and Supplementary Fig. 7). Having removed inferred naïve and iNKT cells, we noted specific areas enriched for Th1, Tfh and T_CM_-like gene expression signatures (Fig. 6D) and canonical genes (Fig. 6E). We also noted, as for PbTIIs, populations of GC Tfh cells expressing high levels of *Cxcr5*, *Bcl6*, and *Pdcd1* and a neighbouring distinct cluster expressing high levels of *Sostdc1* (Fig. 6E). Importantly, prior to re-infection, T_CM_ and Tfh-like transcriptomes appeared as a continuum, with Th1-like transcriptomes exhibiting a degree of separation, thus replicating our observations with PbTIIs (Fig. 6E and F). In contrast to PbTII data, there was a *Foxp3*-expressing cluster with limited clonal connections to other cell-states, consistent with rare instances of iTreg differentiation during primary infection (Fig. 6C and E).

3 days after re-infection, CD11a^hi^ CXCR3^hi^ CD4^+^ T cells continued to exhibit heterogeneous states that were clonally related to each other, both within and between clusters (Fig. 6C). In particular, we noted strong gene expression signatures for Th1, Tfh, GC Tfh and *Sostdc1*^*+*^ GC Tfh cells, as well as rarer clonal links with *Foxp3-*expressing cells (Fig. 6C and D). These data suggest that naïve CD4^+^ T cells gave rise to multiple cell-states during recall within individual clones populating Th1, Tfh and *Sostdc1*^*+*^ Tfh states. Crucially, a comparison of transcriptomic states prior to and after re-infection suggested, as with PbTIIs, that Th1 cells changed substantially while GC Tfh states remained unaltered (Fig. 6F), although the relative frequency of *Sostdc1*^*+*^ to *Sostdc1*^−^ GC Tfh cells changed after re-infection (Fig. 6E and F). Based on proximity within UMAPs, we inferred those cells with the strongest T_CM_ phenotype prior to re-infection, likely adopted a moderate Tfh-like signature after re-infection, with upregulation of *Cxcr5* and *Bcl6* and loss of *Ccr7, Sell, Klf2* (Fig. 6E and F). Finally, the number of differentially expressed genes for each inferred cell-type prior to versus 3 days after re-infection was substantially higher for Th1-memory cells than T_CM_-like cells, with GC Tfh cells being entirely refractory to change over this time-period (Fig. 6G). Tregs also showed limited transcriptional change during recall (Fig. 6G; Supplementary Data 6). Thus, similar to PbTIIs (Fig. 6G; Supplementary Data 7–9), polyclonal GC Tfh clones remained largely refractory during re-infection with malaria parasites, T_CM_/Tfh-like cells exhibited a modest non-proliferative response, and Th1-memory cells exhibited a substantial proliferative effector response.

**Fig. 7:**
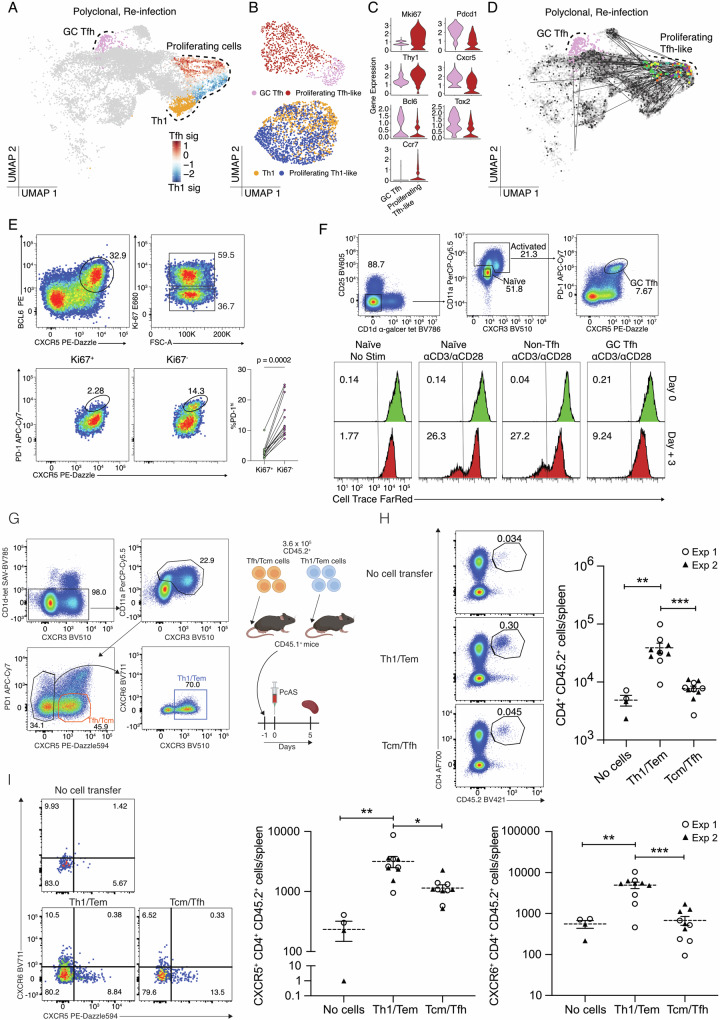
TCR diverse polyclonal GC Tfh cells lack the capacity to proliferate upon re-infection. **A** UMAP of CD11a^hi^CXCR3^+^ polyclonal CD4^+^ T cells 3 days post-re-infection with cell states depicted by colours and dotted boundaries. Proliferating cells show Tfh/Th1 signature scores. **B** UMAP of proliferating Tfh-like cells and GC Tfh cells 3 days post-re-infection (Top) and UMAP of proliferating Th1-like cells and Th1 cells (Bottom) after regressing out G2M phase and S phase signature scores. **C** Violin plots showing expression of genes associated with proliferation (*Mki67*), GC Tfh (*Pdcd1*, *Thy1*—expressed at a low level in GC Tfh cells), Tfh (*Cxcr5*, *Bcl6*, and *Tox2*), and T_CM_ (*Ccr7*). **D** UMAP of CD11a^hi^ CXCR3^+^ polyclonal CD4^+^ T cells (with 5% spike-in of CD11a^lo^CXCR3^-^ naïve cells) showing GC Tfh cells highlighted in pink and TCR sharing (straight edges) from those proliferating Tfh-like cells (coloured by mouse of origin; *n* = 5 mice). **E** Gating strategy to identify proliferating and non-proliferating Tfh-like cells (Top). FACS plots and scatter plot of PD-1 expression on Ki67^+^ and Ki67^-^ Tfh-like cells (Bottom). FACS plots are representative of three independent experiments, with at least four mice per experiment (n = 13 total). Wilcoxon matched-pairs signed rank test was used to assess difference in PD-1 expression between Ki67^+^ and Ki67^−^ populations pooled from three independent experiments. **F** Sorting strategy for isolated T cell subsets from anti-malarial treated mice following infection with *Pc*AS (Top). Representative histograms (from two independent experiments) showing CellTrace^TM^ Far Red intensity in naïve (no stimulation), naïve (anti-CD3/anti-CD28 stimulation), non-Tfh (anti-CD3/anti-CD28 stimulation), and GC Tfh (anti-CD3/anti-CD28 stimulation) populations prior to and 3 days post-stimulation (Bottom). **G** Experimental design: antigen-experienced T_CM_/Tfh-like or non-Tfh cells (harbouring Th1-like Tem cells) were sorted from infected/drug-treated mice at 28 days post-infection. 360,000 cells were transferred into separate groups of naïve, congenically marked recipient mice, which were infected the following day. Spleens were assessed 5 days later. **H** Representative FACS plots showing gating strategy to detect CD4^+^ CD45.2^+^ cells, including a “no cell transfer control”, and scatter plots of numbers of splenic CD4^+^ CD45.2^+^ cells; one-way ANOVA with Šídák’s multiple comparison testing; ***P = 0.0008; **P = 0.0051. **I** Representative FACS plots and gating for assessment of CXCR5 and CXCR6 expression by CD4^+^ CD45.2^+^ cells, and corresponding scatter plots of numbers of CXCR5^+^ or CXCR6^+^ CD4^+^ CD45.2^+^ cells. Scatter plots show data from 2 independent experiments (*n* = 2/experiment for “no-cell transfer”; *n* = 5/experiment for other groups). Bars represent mean ± SEM; one-way ANOVA with Šídák’s multiple comparison testing, CXCR5 plot: *P = 0.0137, **P = 0.0068; CXCR6 plot **P = 0.0020, ***P = 0.0001.

Although polyclonal GC Tfh cells appeared refractory and non-proliferative during re-infection, we considered the possibility that initiation of cell division by some of these cells over the 3-day period had been missed due to infrequent scRNA-seq sampling. Indeed, while proliferating PbTIIs had exhibited no obvious Tfh-like phenotype (Fig. 3A), proliferating polyclonal CD4^+^ T cells did contain a subset expressing *Cxcr5* and *Bcl6*, not *Cxcr6* (Figs. 6E and 7A), which was partially consistent with a Tfh-like phenotype, although *Ccr7* expression was also noted (Fig. 6E). To determine if these cells were GC Tfh-like, we regressed out cell cycle genes, and compared against GC Tfh cells from the same timepoint (Fig. 7B). Proliferating *Cxcr5*^*+*^ cells exhibited transcriptomes distinct from GC Tfh cells, while comparator analysis of Th1 versus proliferating Th1 cells during re-infection revealed extensive transcriptional similarity (Fig. 7B). Proliferating *Cxcr5*^*+*^ cells exhibited lower levels of *Bcl6, Cxcr5, Tox2*, and *Pdcd1*, and higher levels of *Ccr7* and *Thy1* (encoding CD90, reported as downregulated on GC Tfh cells^38^) than GC Tfh (Fig. 7C). Moreover, there was no TCR sharing between proliferating *Cxcr5*^*+*^ cells and GC Tfh cells (Fig. 7D). Taken together our data suggest proliferating *Cxcr5*^*+*^ cells had not derived from GC Tfh. To further test our hypothesis that GC Tfh cells are  non-proliferative during re-infection, we examined Tfh-like cells by flow cytometry (based on BCL6/CXCR5 co-expression) 3 days after re-infection. Although Ki67^+^ and Ki67^−^ Tfh-like cells were observed, PD-1^hi^ GC Tfh cells were substantially less prevalent in the proliferating Ki67^+^ compartment (Fig. 7E). These data revealed that GC Tfh cells, unlike other Tfh-like cells, had not proliferated during re-infection. This raised the question of whether GC Tfh was intrinsically incapable of proliferating during re-infection. To test this, we sorted GC Tfh, or antigen-experienced non-Tfh and naïve counterparts from mice after primary infection and treatment, and assessed their proliferation after in vitro TCR stimulation. Compared to naïve or non-Tfh memory controls, GC Tfh remained viable but failed to proliferate over a three-day period of TCR stimulation (Fig. 7F).

Next, we tested the cell-intrinsic potential of Tfh/T_CM_-like and Th1-like cells to proliferate in vivo. We sorted CD11a^hi^ CXCR3^+^ CXCR5^−^ (Th1-like T_EM_ cells) and CD11a^hi^ CXCR5^+^ PD-1^−^ (Tfh/T_CM_-like cells) from infected and drug-treated mice, at 28 days p.i., and transferred equal numbers of these into congenically marked naïve recipients (Fig. 7G), followed by infection and assessment of spleens 5 days later. Importantly, the progeny of transferred Th1/T_EM_-like cells were readily detected above the background, while those of Tfh/T_CM_-like counterparts were not detected above the background (Fig. 7H). Further assessment of surface phenotype suggested that T_CM_/Tfh-like cells may have given rise to progeny with expression of CXCR5, yet cell numbers were lower than those generated from Th1-like T_EM_ cells. (Fig. 7I). Taken together, our in vitro and in vivo data suggested that during re-infection with malaria parasites, pre-existing polyclonal GC Tfh and Tfh/T_CM_-like cells are intrinsically less capable of mounting a proliferative response than Th1-like T_EM_ cells.

Finally, having confirmed similarly heterogeneous responses of PbTII and polyclonal CD4^+^ T cells 3 days after re-infection, we sought to test whether early upregulation of ~400 genes associated with RNA processing in PbTII cells, including ribosome biogenesis and splicing (Fig. 4C), could be detected as a global increase in protein translation in polyclonal CD4^+^ T cells early after re-infection. Using puromycin, which as a tRNA mimic binds to active ribosomes, we observed that although antigen-experienced, polyclonal CD4^+^ T cells were translationally active prior to re-infection compared to naïve counterparts, this did not increase 24 h after re-infection (Supplementary Fig. 8A). Further examination of antigen-experienced, cellular phenotypes indicated translational activity amongst GC Tfh, Tfh-like and non-Tfh cells prior to re-infection, the proportions of which did not alter early after re-infection (Supplementary Fig. 8B).

Taken together, our data indicated that heterogeneous responses had occurred amongst polyclonal CD4^+^ T cells during re-infection with malaria parasites, with GC Tfh and Tfh-like cells being largely refractory. In contrast, Th1-like cells responded quickly and then proliferated, but did so without a pronounced early increase in protein production.

### Th1-memory clones exhibit a robust proliferative Tr1 recall response during re-infection

**Fig. 8:**
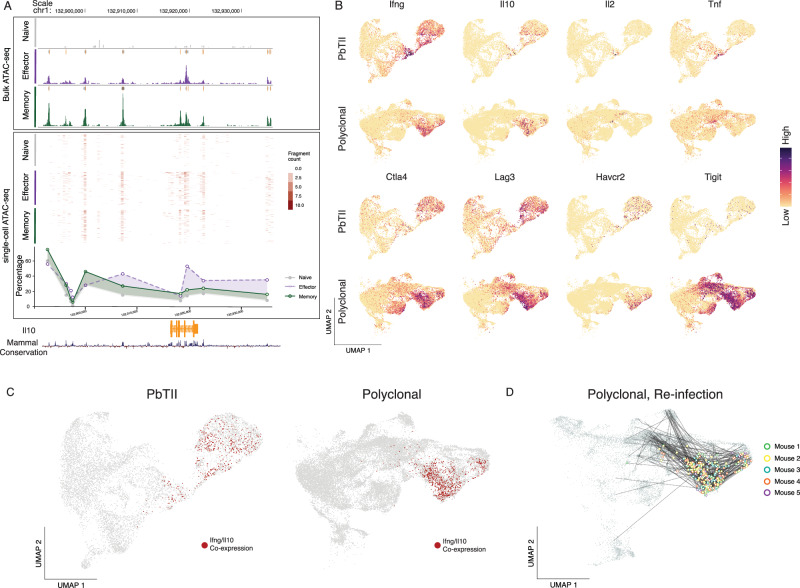
Th1-memory clones exhibit a robust proliferative Tr1 recall response during re-infection. **A** UCSC genome browser tracks displaying genomic accessibility signals around the *Il10* gene locus. (Top) Mean bulk ATAC-seq genome coverage. Boxes at the top of each coverage represent peaks called using MACS2. Data representative of two independent experiments showing similar results^23^. (Middle) Accessibility profiles of 200 randomly selected single cells from plate-based single-cell ATAC-seq data^23^. (Bottom) Percentage of cells with open accessibility at each genomic region **B** UMAP of PbTII cells and polyclonal CD4^+^ T cells showing mRNA expression of *Ifng*, *Il10*, *Il2*, *Tnf*, *Ctla4*, *Lag3*, *Havcr2*, and *Tigit*. **C** UMAP of PbTII cells (Left) and polyclonal CD4^+^ T cells (Right) with *Il10* and *Ifng* co-expressing cells highlighted in red. **D** UMAP of CD11a^hi^CXCR3^+^ polyclonal CD4^+^ T cells (with 5% spike-in of CD11a^lo^CXCR3^−^ naïve cells) showing TCR sharing (straight edges) from those cells co-expressing *Il10* and *Ifng* (highlighted in open circles and coloured by mouse of origin; *n* = 5 mice).

Given that multiple exposures to *Plasmodium* parasites in children have been associated with a switch within antigen-experienced CD4^+^ T cells from the production of pro-inflammatory cytokines TNF, IFN-γ and IL-2 to immunoregulatory IL-10 by a Tr1 state^11^, we lastly sought to examine Tr1 transcriptional dynamics during re-infection in mice. Firstly, analysis of chromatin accessibility via bulk ATAC-seq of PbTIIs, generated in our previous study^23^, suggested increased accessibility around the *Il10* locus at memory stages compared to naïve states (Fig. 8A). This was corroborated by plate-based single-cell ATAC-seq, in which a greater proportion of PbTII cells displayed accessibility around the *Il10* locus at memory stages than at naïve (Fig. 8A). Together, these data suggested *Il10* might be heterogeneously upregulated by CD4^+^ T cells during re-infection. Indeed, we noted both PbTII cells and polyclonal CD4^+^ T cells displayed robust and prolonged co-expression of *Il10* and *Ifng* during re-infection, consistent with their definition as Tr1 cells in murine malaria (Fig. 8B and C). In contrast, *Il2 and Tnf* were only transiently upregulated by PbTII cells during re-infection, (Fig. 8B). Furthermore, both PbTII and polyclonal CD4^+^ T cells upregulated immune checkpoint or inhibitory molecules, *Ctla4, Lag3, Havcr2* and *Tigit* upon recall (Fig. 8B). Thus, our data suggested that primary infection triggers Th1-memory cells that do not sustain TNF or IL-2 production upon re-infection, and instead exhibit a prolonged Tr1 recall response. Furthermore, TCR clonal analysis of polyclonal CD4^+^ T cells suggested clonal links of Tr1 cells to other cell states including T_CM_/Tfh-like cells and *Sostdc1*^*+*^ GC Tfh (Fig. 8D). Therefore, single naïve CD4^+^ T cells gave rise to multiple states during recall including *Sostdc1*^*+*^ GC Tfh that remain refractory during re-infection and Tr1 cells that readily re-express IFN-γ and IL-10.

## Discussion

In this study, we sought to understand how antigen-specific CD4^+^ T cells, activated and positioned in the spleen after primary experimental malaria in mice, responded thereafter to a second infection. In doing so, we aimed to model the experiences of children living in malaria-endemic regions, who are exposed to sequential infections over short periods of time. Although some of their circulating CD4^+^ T cells appear to become increasingly immunoregulatory over multiple exposures (as inferred from antigenic re-stimulation in vitro leading to IL-10 production^11^), how this relates to all antigen-experienced CD4^+^ T cells in secondary lymphoid tissues remains unclear. Moreover, although CD4^+^ T-cell dependent, isotype-switched, affinity-matured IgG is a cardinal mechanism of adaptive immunity to malaria^39^, the effect of re-infection on ongoing Tfh and other CD4^+^ T-cell responses is not fully understood. However, a previous report by Latham et al. examined polyclonal CD4^+^ T cell responses during re-infection with *P. chabaudi*, and noted that Th1-like memory cells increased in number, while GC Tfh and Tfh-like cells did not^18^. This suggested heterogeneity amongst antigen-experienced, *Plasmodium-*specific CD4^+^ T cells, with Tfh-like cells potentially being refractory compared with Th1-like counterparts, although antigen-specific T cells were not directly assessed. In our mouse model, a complex spectrum of transcriptional states and micro-anatomical locations was observed in CD4^+^ T cells after primary infection, even for those of a single specificity. This highlights that the acquisition of multiple states by a single T-cell clone is common in this model. Whether certain TCR sequences pre-dispose towards GC Tfh or Th1-memory remains to be determined, but based on other model systems^40–43^, it seems likely that the strength and duration of interactions with professional and non-professional antigen-presenting cells plays a role in giving rise to such a complex landscape of GC Tfh, Th1-memory, and other fate choices.

During re-infection, which elicited a transient but readily controlled pathogen load, we noted unexpectedly different recall responses by the heterogeneous, antigen-experienced CD4^+^ T cells, consistent with previous work^18^, even when T cells were of the same antigen-specificity, located within the same organ, in either similar or distinct microanatomical regions. For example, T_CM_-like and Th1-memory cells were both located in splenic T-cell zones, and yet displayed dramatically different responses, with T_CM_-like cells, but not Th1-memory cells failing to proliferate over a three-day period. This suggests a fundamental difference between Th1-memory and T_CM_-like cells in their ability to respond to antigenic stimulation in vivo, which was confirmed here by adoptive transfer into and infection of naïve hosts. It is remarkable that T_CM_-like cells, considered a prime mediator of immunological memory, did not appear to enter a proliferative phase during the period of assessment, nor mounted a substantial immune effector response. It is possible, however, that a longer period of analysis, as performed in a recent study^18^, may be necessary to clearly observe the progeny of T_CM_-like cells.

Of note, a Tfh-like proliferating phenotype did emerge amongst polyclonal cells during re-infection of immune hosts, which raises the question of where these cells arose from and why were such cells not detected amongst PbTII cells or in naïve hosts receiving T_CM_/Tfh-like cells. One possibility is that these cells arose from naïve T cells primed for the first time during re-infection. Another hypothesis is that PbTII cells do not capture the full range of responses that polyclonal CD4^+^ T cells exhibit. Future work is needed to determine the origin of proliferating Tfh-like cells during re-infection.

The capacity of GC Tfh and Tfh-like cells to respond a second time remains unclear during parasitic infection. However, in other systems, such as viral infection or vaccination, proliferative responses and functional plasticity have been reported^15–17^. It is unclear why we and others^18^ have not observed GC Tfh and Tfh-like cells to respond during secondary challenge in malaria. Persisting infection is an unlikely reason since mice were treated with antimalarial drugs and adoptive transfer into naïve mice removed any infection-induced effects. Hence, we hypothesise that GC Tfh and Tfh-like cells induced during parasitic infection are fundamentally different from those elicited during viral infections or vaccination. Future experiments will be needed to test this directly.

In our model, GC Tfh cells appeared completely refractory to transcriptional change for 3 days during re-infection, neither proliferating nor exhibiting an immune effector recall response. The persistence of GC Tfh cells 4 weeks after the initial infection and antimalarial treatment suggests their continued role in affinity maturation of antibodies, which are known to accumulate in circulation over this time-period^21,44–46^. Hence, we reasoned that these GC Tfh were functional at the time when a second infection was experienced. The lack of transcriptional change in these cells could be due to numerous, non-mutually exclusive factors. Firstly, GC Tfh cells might already have been maximally stimulated with antigen provided to them by cognate GC B cells. Secondly, GC Tfh cells might be sequestered away from a bolus of antigen arriving in the spleen after intravenous injection. Given that GC Tfh cells showed limited movement outside of GCs and surrounding follicles^47^ even after rechallenge^48^, it is possible they lack exposure to intact antigens. Thirdly, GC Tfh may be inherently refractory to proliferation, as indicated by our in vitro stimulation experiments. While others have reported on the lack of ongoing proliferation of GC Tfh cells during a primary immune response^38,49^, further studies are warranted on the refractory nature of persisting GC Tfh cells during secondary immune responses.

In stark contrast to GC Tfh cells, we observed that rapid and dynamic changes occurred to Th1-memory cells during re-infection. As expected, Th1-memory cells initiated a wave of immune effector molecule production, including IFN-γ and IL-10, prior to a transcriptional programme of cellular proliferation. This confirms, in our system that Th1-memory cells exert function prior to dividing. This raises the question of why a Th1-memory cell needs to proliferate at all if immune function has already been initiated. One hypothesis is that Th1-memory cells have evolved the dual features of rapid function and amplification of their responses through division. Given that cellular proliferation requires a set period of time, one might speculate on the benefit of immune function being initiated first, to be followed later by proliferation. In addition, our scRNA-seq-based, temporally-informed transcriptional network analysis indicated that no transcriptional links existed between the transcription factor hubs that controlled effector function versus proliferation. Hence, we propose that during re-infection, the processes controlling the magnitude or quality of recall have no bearing on the amplification of this process via cellular proliferation, the implication being that whatever response is triggered, Th1-memory cells will be subject to proliferative amplification.

In addition, a dynamic related to mRNA processing appears to emerge alongside that of immune effector function in Th1-memory cells prior to cellular proliferation. These same genes had been upregulated during primary Th1 differentiation, but only as cells progressed through cell division. We reasoned that early production of hundreds of proteins, themselves associated with converting mRNA into protein, might be detectable as a global increase in protein production early during Th1-recall. This was not the case, however, since protein translation, in fact, dropped slightly in polyclonal CD4^+^ T cells early after re-infection. Therefore, although transcription of genes needed for mRNA processing may be initiated early during Th1-recall, we speculate this is not required for immediate immune function, and instead prepares the cell for subsequent proliferation 24–48 h later.

Our study sought to examine the similarities and differences between the peak Th1 response in primary responses versus re-infection. By focussing our attention on “peak” effector responses, we excluded any genes involved in cellular proliferation. In doing so, we noted that only ~60 genes were upregulated upon Th1-recall. This set included many genes associated with primary Th1 responses including *Irf4, Irf8, Havcr2, Tnfsf4, Il2ra, Ctla4, Ifng, Il10, Ccl3, Ccl4*, and *Gzmb*. In addition, ~600 genes were stably maintained by Th1-memory cells compared to naïve counterparts. Many of these genes have yet to have functions ascribed during either primary or secondary immune responses. Our study revealed no genes were uniquely upregulated in recall compared to primary Th1 responses and also confirmed that genes used by many immunologists to measure recall, including OX40, CD25, and the cytokine IFN-γ were likely the best choices for examining this phenomenon. An important ramification of our genome-scale analysis is that Th1-recall is a focussed, partial facsimile of primary Th1 responses. Hence, it is likely that whatever pre-dispositions are set within Th1 cells during an initial malaria infection these are likely to be difficult to shift during re-infection. This raises the hypothesis that primary immune responses may be the primary determinant for the quality of any subsequent responses and that immune interventions should be made before or as a child experiences their first infection with malaria parasites.

Our dynamic analysis also revealed that TNF and IL-2 production were only transiently expressed during recall. Instead, the Tr1 cytokines IL-10 and IFN-γ were robustly expressed even as the cells began to proliferate. This suggests that the Th1-recall responses observed in our mouse model of repeated malaria infection are, in fact, potent Tr1 responses. We reason here that these potent Tr1 proliferative responses serve not only to control parasite numbers but also to do so without triggering unwanted immune pathology. Our data are consistent with those of children living in malaria-endemic regions^11^, suggesting that repeated infections trigger the emergence of Th1-memory cells in the spleen that can mount potent, proliferative Tr1 responses soon after re-infection. Chromatin accessibility assessments confirmed increases in accessibility around the *Il10* locus as a result of a single infection. It remains to be determined whether sequential infections continue to alter the epigenomic profile of CD4^+^ T cells, both at the *Il10* locus and elsewhere. In summary, our data indicate that parasite-specific CD4^+^ T cells acquire a complex spectrum of antigen-experienced states in the spleen during experimental malaria and that re-infection triggers a variety of different responses in these cells. Of note, those CD4^+^ T cells that support antibody-mediated immunity appeared least affected by re-infection, while those associated with pro-inflammatory and immune-regulatory responses were rapidly called upon and their responses amplified through cellular proliferation. Our data highlight that single TCR clones can be mobilised to simultaneously exhibit a range of different phenotypes and recall functions while being located within the same organ. This reveals that during re-infection with malaria parasites, the immune system had already diversified and allocated skills to expanded progeny from primary infection, while the breadth of TCR sequences serves to cater to antigenic diversity. Thus, improved adaptive immunity to malaria may reside not only in embracing antigen diversity but also in sculpting as early as possible the immunological states exhibited by CD4^+^ T cells.

## Methods

### Mice

C57BL/6J were purchased from the Animal Resources Centre (Western Australia) and PbTII and PbTII.nzEGFP mice were bred in-house. All mice were maintained under specific pathogen-free conditions within the Biological Research Facility of the Doherty Institute for Infection and Immunity (Melbourne, VIC, Australia), or within the Animal Facility at QIMR Berghofer Medical Research Institute (Brisbane, Queensland, Australia). All animals used were females at 6–12 weeks old. All animal procedures, including ethically performed CO_2_-mediated euthanasia, were approved by the University of Melbourne Animal Ethics Committee (SLA-1: 1915018 and 10376) and the QIMR Berghofer Medical Research Institute Animal Ethics Committee (approval no. A1503-601M).

### Adoptive transfer of PbTIIs

Naïve spleens from PbTII and PbTII.nzEGFP mice were harvested and homogenised through 70–100 µm cell strainers and the red blood cells (RBCs) were lysed using RBC Lysing Buffer Hybri-Max (Sigma-Aldrich) or Pharm Lyse (BD). CD4^+^ T cells were enriched via Magnetic-Activated Cell Sorting (MACS) using CD4 (L3T4) microbeads (Miltenyi Biotec). In some instances, PbTII cells were labelled with Violet Proliferation Dye 450 (VPD450) (BD) prior to the adoptive transfer. For that, enriched PbTII cells were washed twice in Dulbecco’s phosphate-buffered saline (D-PBS) and stained with VPD450 at a final concentration of 1 µM at 37 °C for 15 min. After incubation, cells were washed in D-PBS and resuspended in cold RPMI containing penicillin and streptomycin (RPMI/PS). Finally, 10^4^ PbTII.nzEGFP cells were injected per recipient mouse into the lateral tail vein prior to the primary infection; 10^4^ CTV-labelled PbTII cells were injected into recipients at 27 days after primary infection.

### Infection

Thawed *Pc*AS-infected blood stabilites from our biobank were used to infect C57BL/6J single passage mice. *Pc*AS-infected RBCs were obtained from the passage mice and parasitized RBCs (pRBCs) were injected into each recipient mouse via lateral tail vein injection. (10^5^ pRBC for primary infection, and 10^7^ pRBC for re-infection)

### Parasitemia assessment

Parasitemia assessment was carried out as previously reported^21^, also described here: blood samples were collected from the tail vein into RPMI containing 5 U/mL of heparin. Diluted blood samples were then stained with Hoechst 33342 (10 µg/mL; Sigma-Aldrich) and Syto84 (5 µM; Life Technologies) at room temperature, for 30 min in the dark. The staining was quenched with 10 times the initial volume using cold RPMI, and percentage of Syto84^+^ Hoechst33342^+^ RBCs determined by flow cytometry.

### Anti-malarial drug treatment

Artesunate (Guilin Pharmaceutical, kindly provided by J. Mohrle) was dissolved in 5% sodium bicarbonate solution at 50 mg/mL to form sodium artesunate and diluted in 0.9% saline (Baxter) to the final concentration of 5 mg/mL. Mice received intraperitoneal injections of 1 mg of sodium artesunate twice daily from day 7 to day 9, once daily from day 10 to day 16 and twice weekly from day 17 to day 24 post-infection. Mice were also treated with pyrimethamine in the drinking water (70 mg/L, Sigma Aldrich) from day 7 until day 24 post-infection.

### Adoptive transfer of antigen-experienced polyclonal cells and infection

Spleens were recovered from anti-malarial drug-treated donor mice at day 28 p.i., homogenised through 70 µm cell strainers and the RBCs were lysed using Pharm Lyse (BD). CD4^+^ T cells were enriched via MACS) using CD4 (L3T4) microbeads (Miltenyi Biotec) prior to sorting. Using a FACS Aria III (BD), Tfh/T_CM_-like cells were sorted as Live, CD4^+^, TCRb^+^, CD1d-α-galcer^−^, CD11a^high^, PD-1^−^, CXCR5^+^ cells; and Th1-like cells were Live, CD4^+^, TCRb^+^, CD1d-α-galcer^−^, CD11a^high^, CXCR3^+^, CXCR5^−^ cells. Finally, 3.6 × 10^5^ cells were injected per naïve recipient mouse, divided into two separate groups: Tfh/T_CM_ and Th1-like. The recipient mice were infected in the following day with 10^6^ pRBCs, and spleens were harvested 5 days later for flow cytometry analysis.

### Flow cytometry

Spleens were harvested in cold RPMI/PS and homogenised through a 70/100 µm cell strainers to create single-cell suspensions, followed by RBC lysis using Lysing Buffer Hybri-Max (Sigma-Aldrich) or Pharm Lyse (BD). Cells were then stained with Zombie Yellow viability dye (Biolegend) or Live/Dead aqua (Invitrogen), followed by Fc receptor block (BD). Finally, cells were stained with titrated panels of monoclonal antibodies (Supplementary Data 10) diluted in PBS containing 1% of FCS and 2 mM EDTA and samples were incubated for 20 min on ice in the dark. For intracellular staining, Foxp3/Transcription Factor Staining Buffer Set (eBiosciences) was used to fix and permeabilise cells prior to staining with panels of monoclonal antibodies (Supplementary Data 10) for 30 min on ice in the dark. Finally, cells were washed and acquired on Fortessa cytometer (BD). All data was analysed using FlowJo (Treestar 10.8.0).

### In vitro proliferation assay of antigen-experienced polyclonal cells

MACS enriched CD4^+^ T cells from five pooled splenocytes were sorted for three distinct populations: naïve (CD25^−^, CD1d- α-galcer^−^, CXCR3^−^, CD11a^lo^), non-Tfh (CD25^−^, CD1d-α-galcer^-−^, CXCR3^+^, CD11a^hi^, CXCR5^−^, PD-1^−^), and GC Tfh (CD25^−^, CD1d-α-galcer^−^, CXCR3^+^, CD11a^hi^, CXCR5^−^, PD-1^−^) using Cytoflex SRT (Beckman Coulter). Sorted cells were labelled with 1 µm of CellTrace Far Red (CTFR) at room temperature for 20 min. Cells were counted and then added onto plates bound with anti-CD3 (10 µg/mL) and anti-CD28 (5 µg/mL) (concentrations chosen after titration to minimise activation-induced cell death) and cultured in complete RPMI supplemented with 100 U/mL IL-2 and 1 µM p2x7r inhibitor (A-438079; Santa Cruz Biotech). Cells were analysed at set time points for dilution of CTFR.

### Puromycin staining

Following RBC lysis, splenocytes were incubated in RPMI, containing 10% foetal calf serum, penicillin/streptomycin and puromycin at 1 ng/mL for 45 min at 37 °C. Cells were washed three times in PBS, stained with Live/Dead marker and surface staining, washed, fixed and permeabilized using BD Cytofix/Cytoperm kit. Permeabilized cells were incubated with mouse anti-puromycin (Sigma-Merck, at 1:10,000) for 1 h on ice, washed twice with permeabilization buffer and incubated with anti-mouse IgG2aκ AF488-conjugated antibody for 1 h, on ice. Cells were washed twice and acquired on Fortessa cytometer (BD). All data analysed using FlowJo (Treestar 10.8.0).

### Cell sorting and scRNA-seq

#### PbTIIs

Spleens were harvested and homogenised through 100 µm cell strainers to create single-cell suspensions. RBCs were lysed using Lysing Buffer Hybri-Max (Sigma-Aldrich). Samples were then washed twice in PBS containing 0.5% BSA and 2 mM EDTA, and CD4^+^ T cells were enriched using MACS CD4 (L3T4) microbeads (Miltenyi Biotec). Next, enriched cells were prepared for cell sorting by Fc receptor blocking and stained with titrated monoclonal antibodies (Supplementary Data 10). Finally, cells were washed and re-suspended in PBS 2% BSA containing propidium iodide at dilution of 1:500. PbTII cells (CD4^+^ TCRVα2^+^ TCRVβ12^+^) were sorted using a FACS Aria III (BD) into a 1% BSA/PBS buffer. After sorting, PbTIIs were loaded onto the Chromium controller and cDNA-sequencing libraries were prepared using Single-cell 3’ reagent kits (10x Genomics).

#### Polyclonal CD4^+^ T cells

Spleens were harvested and homogenised through 70 µm cell strainers to create single-cell suspensions. RBCs were lysed using BD Pharm Lyse (BD). Next, samples were washed and incubated with Fc receptor-blocking antibodies followed by titrated monoclonal antibodies and TotalSeq-C (BioLegend) mouse hashtags (Supplementary Data 10) for 20 min on ice each, in the dark. Finally, cells were washed twice, resuspended in PBS 2% BSA containing propidium iodide at dilution of 1:500. For each timepoint, samples from five individual mouse replicates were pooled together and live, “naïve” TCRβ^+^ CD4^+^ CXCR3^−^ CD11a^lo^ and “experienced” TCRβ^+^ CD4^+^ CXCR3^+^ CD11a^hi^ T cells were sorted using a FACS Aria III (BD) into a 2% BSA/PBS buffer. After sorting, “naïve” and “experienced” cells were pooled at a 1:9 ratio, washed and resuspended in 0.04% BSA/PBS buffer, and loaded onto the chromium controller and cDNA-sequencing libraries were prepared using Chromium GEM-X Single Cell 5’ Kits v3 (Catalogue #1000695;10x Genomics).

### Processing of data

#### PbTII dataset

Cell Ranger v3.0.2 (*“cellranger count”*) was used to process 10x Genomics gene expression FASTQ files with 10x Genomics mouse genome v2020-A and *egfp* sequence (accession number: EU05636.1) as reference.

#### Polyclonal dataset

Cell Ranger v6.1.1 (*“cellranger multi”*) was used to process 10x Genomics gene expression, VDJ T cell receptor α/β, and mouse-specific hashtag oligos (HTO) FASTQ files with 10x Genomics mouse genome v2020-A and refdata-cellranger-vdj-GRCm38-alts-ensembl-5.0.0 as references.

### Quality control of scRNA-seq data

#### PbTII dataset

Only genes expressed in 3 or more cells were considered. Cells outside the thresholds of 200–5000 expressed genes and up to 15% mitochondrial content were removed. High-quality transcriptomes of 2672 naïve PbTII cells and 5423 antigen-experienced PbTII cells were considered for analysis shown in Fig. 2 (Supplementary Fig. 9A). Naïve PbTII cells were marked by lack of *egfp* expression and antigen-experienced PbTII cells were marked by *egfp* expression (Supplementary Fig. 9B). From the combined dataset of PbTII cells prior to, and at 1, 2, and 3 days after re-infection, cluster 9 was further identified as a poor-quality cluster and removed from analysis given its low number of detected UMIs and genes and yet appearing as highly proliferative group of cells based on G2M score (Supplementary Fig. 10A), further substantiated by integration using *single-cell variational inference* (*scVI*) from scvi-tools package (v0.17.1)^32^, which placed cluster 9 with other highly proliferative cells (clusters 3 and 5) (Supplementary Fig. 10B). Altogether high-quality transcriptomes of 9245 antigen-experienced PbTII cells (marked by *egfp* expression) were proceeded to downstream analysis (Supplementary Fig. 10C).

#### Polyclonal dataset

Only genes expressed in 3 or more cells were considered. TCR genes (TRAV-/TRBV-/TRAJ-/TRBJ-) were also removed from the dataset. Cells outside the thresholds of 300–6000 expressed genes and up to 10% mitochondrial content were removed. Cells identified as doublets were removed by demultiplexing based on HTO using “HTODemux()” function from *Seurat* package (v4.1.0)^50^. Clusters of cells expressing high levels of *Cd74* and *Cd8a* were identified as non-CD4^+^ T cells were removed. A cluster showing enrichment of *Trav11-Traj18* and *Zbtb16* marking iNKT-cells was removed^37^. Altogether high-quality transcriptomes of 28,296 polyclonal cells were proceeded to downstream analysis.

### Normalisation, feature selection, and scaling

Normalisation of data, selection of highly variable features, and scaling of data were performed using “SCTransform()” function from *Seurat* package with default settings.

### Dimensionality reduction

Using the Pearson residuals of all highly variable genes computed from *Seurat*’s “SCTransform()”, principal component analysis (PCA) was performed using “RunPCA()” function from *Seurat* package, and the computed principal components (PCs) as inputs to perform uniform manifold approximation and projection (UMAP) using “RunUMAP()” function from *Seurat* package.

### Unsupervised clustering

Unsupervised clustering was performed using “FindNeighbors()” function followed by “FindClusters()” function from *Seurat* package.

### Gene signature scoring

Signature scores were computed for each cell in the dataset using “AddModuleScore()” function from *Seurat* package (gene lists for Th1, Tcm, and Tfh signatures provided below).

### Data integration

To perform the integration of PbTII datasets highlighted in Fig. 1 and Supplementary Fig. 1, we used two different approaches: (1) Using *Seurat* package, individual dataset was first processed using “SCTransform()”. Datasets were then merged together and “FindIntegrationAnchors()” function was used to identify anchors across datasets, and finally “IntegrateData()” function was used to integrate data. The integrated assay was used to run PCA and UMAP to generate UMAP cell embeddings representing the integrated space. (2) Using “RunHarmony()” function from *Harmony* package (v0.1.0)^24^, data was integrated, followed by running PCA and UMAP using the *Seurat* package to generate UMAP cell embeddings representing the integrated space. All parameters were kept as default. Each experiment was identified as a separate batch.

To perform the integration of PbTII datasets highlighted in Supplementary Fig. 10 and Fig. 5, we used *scVI*. Prior to the integration of Th1-recall cells with Th1-like cells from our previous study^23^ highlighted in Fig. 5, D0, D7 p.i., and D28 p.i. PbTII cells from our previous study were analysed (Supplementary Fig. 11A) and Th1 signature was visualised (Supplementary Fig. 11B) to identify and segregate clusters of cells with the highest Th1 signature (Supplementary Fig. 11C). *scVI* was also used to integrate PbTII and polyclonal datasets highlighted in Supplementary Fig. 12. All parameters were kept at default except 2 hidden layers were used for encoder and decoder neural networks, the negative binomial model used, and the number of epochs set to *n* =(1,000,000/total number of cells) to train the model. Latent variables were used as input to run UMAP using *Scanpy* package (v1.9.1)^51^.

### Pseudotime inference and transcriptome dynamics analysis

Pseudotime was inferred based on latent variable 1 coordinates from Bayesian Gaussian Latent Variable Modelling (BGPLVM) dimensionality reduction using *GPfates*^22^. Automatic expression histology (AEH) from *spatialDE* (v1.1.3)^28^ was applied to perform transcriptomic dynamics analysis using parameters number of patterns (*c*) = 9 and lengthscale = 0.4, where *c* specifies the number of gene groups, with each group displaying distinct expression dynamics along pseudotime. Only significantly variable genes along pseudotime were considered (FDR < 0.05).

### Differential gene expression analysis

Differential gene expression analysis was performed using “scvi.differential_expression()” function from *scVI*. All expressed genes were considered in generating the model. The following comparisons were done for comparing transcriptomes before and after re-infection: (1) cluster 12 (Th1-memory cells before re-infection) versus cluster 8 (Th1-recall cells after re-infection) from PbTII dataset, (2) cluster 2 (Tcm/Tfh-like cells before re-infection) versus cluster 10 (Tcm/Tfh-like cells after re-infection) from PbTII dataset, (3) cells before re-infection from cluster 9 versus cells after re-infection from cluster 9 (GC Tfh cells) from PbTII dataset, (4) cluster 9 (Th1-memory cells before re-infection) versus cluster 12 (Th1-recall cells after re-infection) from polyclonal dataset, (5) cluster 0 (T_CM_-like cells before re-infection) versus cluster 8 (Tcm-like cells after re-infection) from polyclonal dataset, (6) cells before re-infection from cluster 16 versus cells after re-infection from cluster 16 (GC Tfh cells) from polyclonal dataset (Fig. 3B for (1–3) and Fig. 6G for (4–6)). Clusters from PbTII and polyclonal datasets are shown in Supplementary Fig. 12A. Data integration of PbTII and polyclonal datasets was performed to ensure similar transcriptomes were compared in this analysis. Th1, Tcm, and Tfh signatures were visualised on integrated UMAP of PbTII and polyclonal datasets to compare clusters of similar phenotypes (Supplementary Fig. 12B). The following comparisons were done to define Th1 response: (1) clusters 5 and 10 (naïve cells) versus cluster 3 (Th1-primary cells) from droplet-based scRNA-seq generated from previous study^23^, (2) clusters 5 and 10 (naïve cells from previous study^23^) versus cluster 8 (Th1-recall cells from PbTII dataset), and (3) cluster 3 (Th1-primary cells) versus cluster 7 (Th1-memory cells) (Fig. 5B and C). Clusters of PbTII cells from previous studies and visualisation Th1 signature in each cluster to compare clusters of similar phenotypes are shown in Supplementary Fig. 11C. Genes with Bayes_factor > 3 were considered to be differentially expressed.

### Gene ontology term enrichment analysis

Enrichment analysis was performed using *GOrilla* (*p* < 0.05)^52^. Input gene lists were differentially expressed genes. Enriched GO terms were further curated using *REVIGO* to identify unique GO terms based on semantic similarity^53^.

### Spatial transcriptomics

Spatial profiling of transcriptomes from fresh frozen splenic tissue samples was conducted at the Broad Institute using Slide-seqV2^25^. All downstream analyses on spatial transcriptomic datasets were performed as previously described^26^. Briefly, pre-processing and quality control to obtain high-quality datasets were followed by deconvolution to estimate cell-type identities and proportional contributions using *cell2location*^54^. Unbiased clustering was performed on neighbourhood-adjusted library size-normalised data to identify germinal centre regions within splenic sections. When plotting CD4^+^ T cell subsets in Fig. 1E and Supplementary Fig. 2, colour scales were condensed so that any bead with a proportion of these cell types greater than 50% of maximum in the dataset appeared as the highest value in the colour scale. This transformation was not applied when performing cell-cell colocalization inference.

### Bulk ATAC-seq analysis

Analyses were performed as previously reported^23^. Briefly, sequenced reads were mapped to mouse genome MGSCv37 (mm9). Peak calling and quality control were performed. The resulting bigWig tracks and narrowPeak files were visualised using UCSC genome browser (https://genome.ucsc.edu/).

### Single-cell ATAC-seq analysis

Analyses were performed as previously reported^23^. Briefly, sequenced reads were mapped to mouse genome MGSCv37 (mm9) and peak calling was performed. Quality control was performed to obtain a high-quality dataset. Aggregate peak coverage was visualised using UCSC genome browser (https://genome.ucsc.edu/). A gene locus was considered open in accessibility if 1 or more reads were mapped to the region.

### Statistical analyses

Statistical analyses for all flow-cytometry-based protein-expression data were performed using Prism (version 9.1.2 GraphPad software). *P* values are shown as *P < 0.05, **P < 0.01, ***P < 0.001, ****P < 0.0001, unless exact *P* values are indicated.

### Reporting summary

Further information on research design is available in the Nature Portfolio Reporting Summary linked to this article.

### Supplementary information

Supplementary Information

Peer Review File

Description of Additional Supplementary Files

Supplementary Data 1-10

Reporting Summary

### Source data

Source Data

## Data Availability

All raw sequencing data are available via accession numbers provided: GSE233703: scRNA-seq of mouse Plasmodium-specific TCR-transgenic CD4^+^ T cells prior to and after re-infection https://www.ncbi.nlm.nih.gov/geo/query/acc.cgi?acc=GSE233703. GSE233713: scRNA-seq + TCR-seq of mouse polyclonal CD4^+^ T cells prior to and after re-infection. https://www.ncbi.nlm.nih.gov/geo/query/acc.cgi?acc=GSE233713. GSE234253: Spatial transcriptomics of mouse spleens during malaria convalescence. https://www.ncbi.nlm.nih.gov/geo/query/acc.cgi?acc=GSE234253. All raw sequencing data from our previous publication used within our current study is available via the accession numbers at ArrayExpress: E-MTAB-9317 (scRNA-seq data). https://www.ebi.ac.uk/biostudies/arrayexpress/studies?query=E-MTAB-9317. E-MTAB-9393 (bulk ATAC-seq data). https://www.ebi.ac.uk/biostudies/arrayexpress/studies?query=E-MTAB-9393. E-MTAB-9402 (scATAC-seq data). https://www.ebi.ac.uk/biostudies/arrayexpress/studies?query=E-MTAB-9402. Processed data used in the main figures are available in the Source Data file. All other data are available in the article and its Supplementary files or from the corresponding author upon request. Source data are provided with this paper.
