# Supplementary material for: CD4+ T cells display a spectrum of recall dynamics during re-infection with malaria parasites
Source: Nat Commun. 2024 Jun 28;15:5497. doi: 10.1038/s41467-024-49879-6 (PMC11214622; doi:10.1038/s41467-024-49879-6)
Supplement: Supplementary file 1 — Supplementary Information [file 41467_2024_49879_MOESM1_ESM.pdf]

Supplementary Figures

Supplementary Figure 1: Sorting strategy, quality control and integration of scRNA-seq datasets of PbTII cells 28 days after infection from two independent experiments.

(A) FACS gating strategy for isolation of PbTII cells from spleen at day 28 post-infection and parasitemia during *Pc*AS primary infection in the presence or absence (saline-treated control group) of antimalarial treatment. Data are pooled from 2 independent experiments (n=5/group/experiment).

(B) Violin plots showing the distribution of PbTII cells for number of UMIs, proportion of mitochondrial content, and number of genes.

(C) PCA representation of PbTII cells before and after integration using *Seurat*. Cells coloured by experimental origin.

(D) (Left) UMAP representation of PbTII cells after integration using *Seurat* showing the individual clusters from unsupervised clustering (Louvain algorithm). (Right) Bar plot showing frequency of PbTII cells from each experiment in each cluster.

(E) (Left) UMAP representation of PbTII cells after integration using *Harmony*. Cells coloured by experimental origin. (Middle) UMAP representation showing individual clusters from unsupervised clustering (Louvain algorithm). (Right) Bar plot showing the frequency of PbTII cells from each experiment in each cluster.

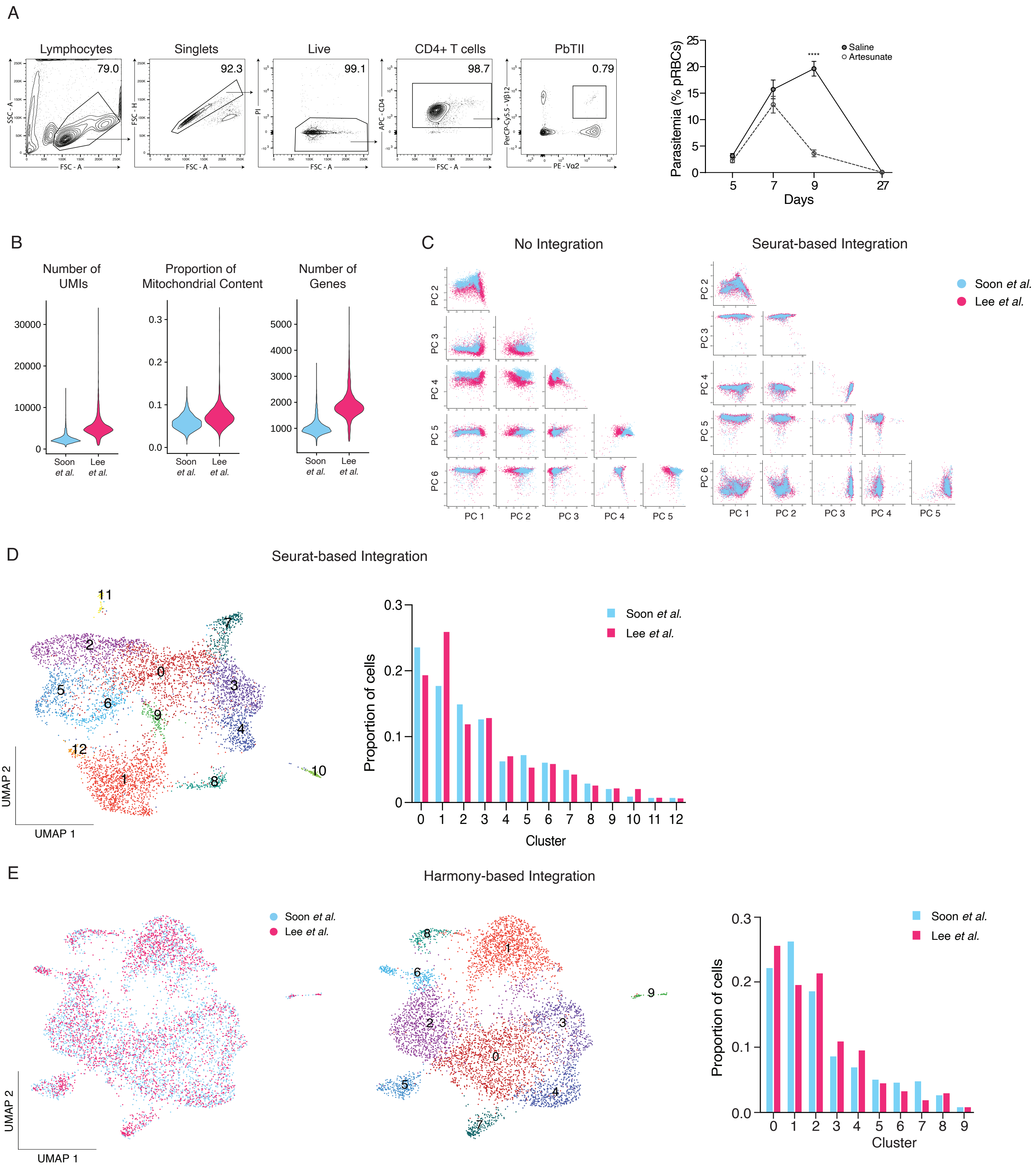
A
Lymphocytes
79.0
Singlets
92.3
Live
99.1
CD4+ T cells
98.7
PbTII
0.79
FSC - A
SSC - A
FSC - H
PI
APC - CD4
PerCP-Cy5.5 - Vβ12
PE - Vα2
Parasitemia (% pRBCs)
Days
Saline
Artesunate
****
B
Number of UMIs
Proportion of Mitochondrial Content
Number of Genes
Soon et al.
Lee et al.
C
No Integration
Seurat-based Integration
PC 1
PC 2
PC 3
PC 4
PC 5
PC 6
Soon et al.
Lee et al.
D
Seurat-based Integration
UMAP 1
UMAP 2
Proportion of cells
Cluster
Soon et al.
Lee et al.
E
Harmony-based Integration
Soon et al.
Lee et al.
UMAP 1
UMAP 2
Proportion of cells
Cluster

Supplementary Figure 2: Spatial maps of mouse spleen tissue generated using Slide-seqV2 reveal GC Tfh transcriptomes in GC B-cell regions.

Spleens were harvested at day 30 post-infection and processed by *Slide-seqV2* and analysed as in Main Figure 1E. Three independent slides are shown here in three columns, with the same GC B cell, cDC2 and follicular B cell data shown in each panel for a given column, with GC Tfh, Th1 memory or Tcm cells shown in red in each row. These slides are replicates for data shown in Figure 1E.

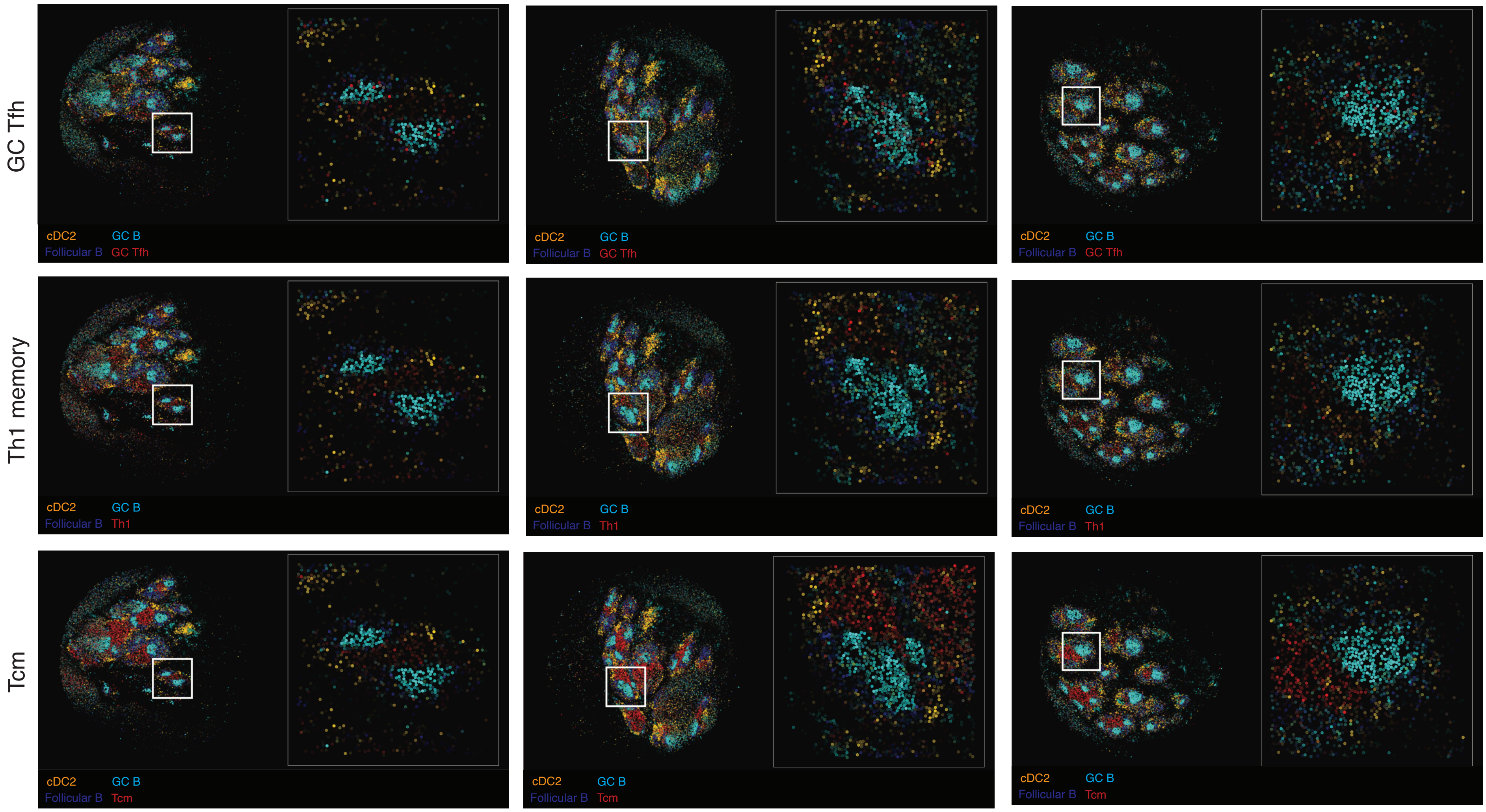

GC Tfh
Th1 memory
Tcm
cDC2
GC B
Follicular B
GC Tfh
Th1
Tcm

Supplementary Figure 3: FACS gating strategy for isolation of antigen-experienced and naïve PbTII cells from spleens at prior to (Top) and 1, 2, and 3 days after re-infection (Bottom).
These data relate to experiments in Main Figures 1 and 2.

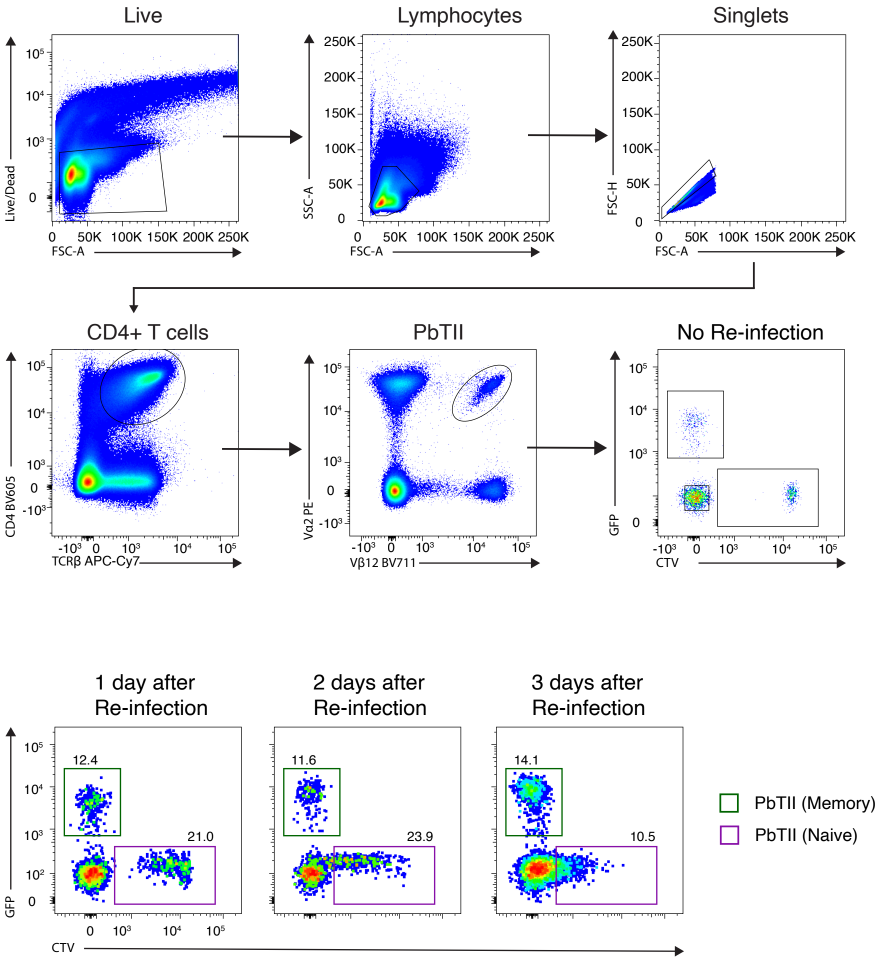

Live
Lymphocytes
Singlets
Live/Dead
FSC-A
SSC-A
FSC-H
CD4+ T cells
PbTII
No Re-infection
CD4 BV605
TCRβ APC-Cy7
Vα2 PE
Vβ12 BV711
GFP
CTV
1 day after Re-infection
2 days after Re-infection
3 days after Re-infection
12.4
21.0
11.6
23.9
14.1
10.5
PbTII (Memory)
PbTII (Naive)

Supplementary Figure 4: Co-expression gene network analysis shows transient link of RNA processing with immune effector function and later with proliferation.

(A) Co-expression network analysis of genes (represented as nodes) in dynamics 2 and 4 using cells from Late pseudotime (in Figure 4A). Nodes are coloured by dynamics of origin (Left) and protein class (Right).

(B) Co-expression network analysis of genes in dynamics 2, 3, 4, and 5 using cells from Mid pseudotime (Left) and Late pseudotime (Right).

Nodes are coloured by dynamics of origin. Edge weight corresponds to Spearman's rho values (only showing rho > 0.2).

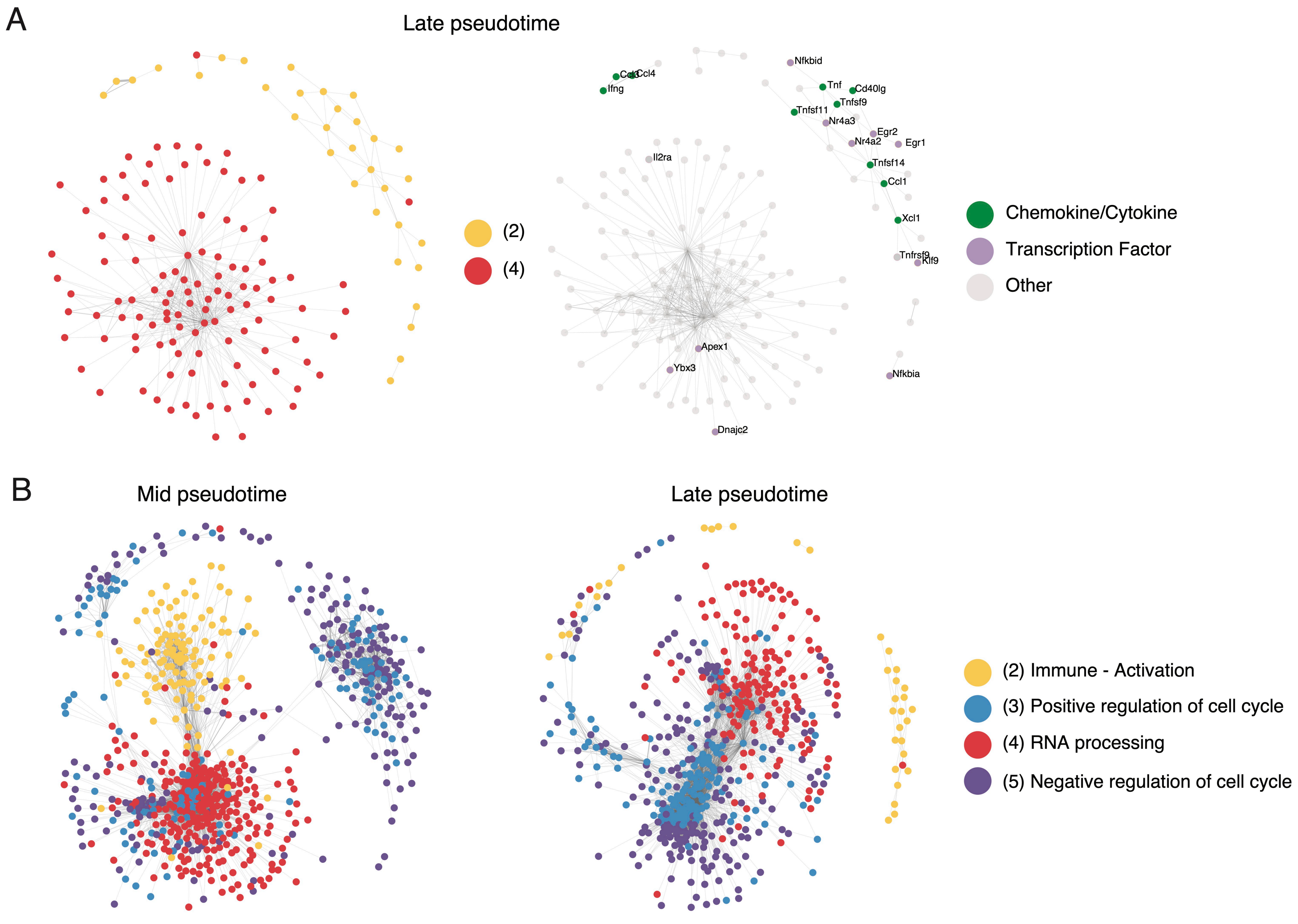
A
Late pseudotime
(2)
(4)
Nfkbid
Ccl3
Ccl4
Ifng
Tnf
Cd40lg
Tnfsf9
Tnfsf11
Nr4a3
Egr2
Nr4a2
Egr1
Il2ra
Tnfsf14
Ccl1
Xcl1
Tnfrsf9
Klf9
Apex1
Ybx3
Nfkbia
Dnajc2
Chemokine/Cytokine
Transcription Factor
Other
B
Mid pseudotime
Late pseudotime
(2) Immune - Activation
(3) Positive regulation of cell cycle
(4) RNA processing
(5) Negative regulation of cell cycle

Supplementary Figure 5: Expression dynamics during memory transition and recall for 1175 genes initially downregulated during primary Th1 differentiation.

Sankey plot schematic showing expression dynamics for the 1175 genes initially downregulated in primary Th1 cells relative to naïve PbTIIs (Up: upregulated, Down: downregulated, Stable: no significant change) at each stage in reference to the preceding stage. Full list of genes downregulated from Th1-memory to recall are shown on the right.

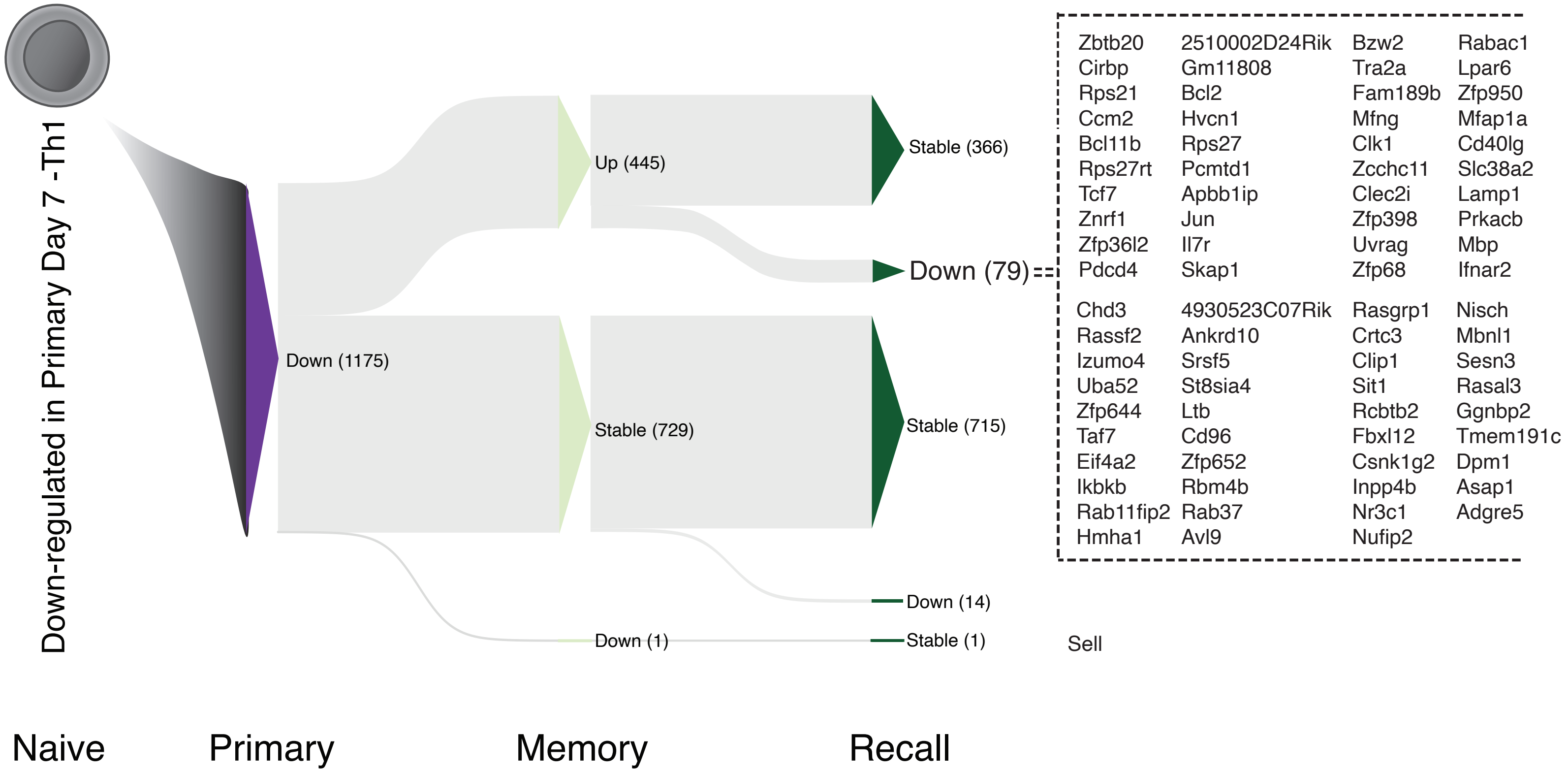

Down-regulated in Primary Day 7 -Th1
Down (1175)
Up (445)
Stable (729)
Down (1)
Stable (366)
Down (79)
Stable (715)
Down (14)
Stable (1)
Zbtb20 2510002D24Rik Bzw2 Rabac1
Cirbp Gm11808 Tra2a Lpar6
Rps21 Bcl2 Fam189b Zfp950
Ccm2 Hvcn1 Mfng Mfap1a
Bcl11b Rps27 Clk1 Cd40lg
Rps27rt Pcmtd1 Zcchc11 Slc38a2
Tcf7 Apbb1ip Clec2i Lamp1
Znrf1 Jun Zfp398 Prkacb
Zfp36l2 Il7r Uvrag Mbp
Pdcd4 Skap1 Zfp68 Ifnar2
Chd3 4930523C07Rik Rasgrp1 Nisch
Rassf2 Ankrd10 Crtc3 Mbnl1
Izumo4 Srsf5 Clip1 Sesn3
Uba52 St8sia4 Sit1 Rasal3
Zfp644 Ltb Rcbtb2 Ggnbp2
Taf7 Cd96 Fbxl12 Tmem191c
Eif4a2 Zfp652 Csnk1g2 Dpm1
Ikbkb Rbm4b Inpp4b Asap1
Rab11fip2 Rab37 Nr3c1 Adgre5
Hmha1 Avl9 Nufip2
Sell
Naive
Primary
Memory
Recall

Supplementary Figure 6: Quality control for scRNA-seq/TCR-seq dataset of CD11a^{hi}/CXCR3^{+} polyclonal CD4^{+} T cells prior to and 3 days after re-infection.

(A) (Top) Ridge plots showing enrichment of hashtag oligos (columns) for negative, singlet (Hashtags 1-5), and doublet groups (rows). Each hashtag represents data from one mouse. (Bottom) Violin plots showing distribution of polyclonal CD4^{+} T cells for number of UMIs, proportion of mitochondrial content, and number of genes detected.

(B) (Top) UMAP of polyclonal CD4^{+} T cells prior to and after re-infection before removal of TCR genes. Cells are coloured by clusters from unsupervised clustering (Louvain algorithm). (Bottom) Dot plots showing expression of TCR genes from top 5 marker genes of each cluster. Colour represents mean normalised expression and size of dots represents percentage of cells expressing these genes.

(C) (Top) UMAP of polyclonal CD4^{+} T cells after removal of TCR genes. Cells coloured by clusters from unsupervised clustering (Louvain algorithm). (Bottom) Violin plots showing distribution of polyclonal CD4^{+} T cells for number of UMIs, proportion of mitochondrial content, *Cd74* expression, and *Cd8a* expression.

(D) UMAP of polyclonal CD4^{+} T cells expressing *Cxcr3* and *Zbtb16* after removal of clusters with high *Cd74* and *Cd8a* expression (shown in **(C)**).

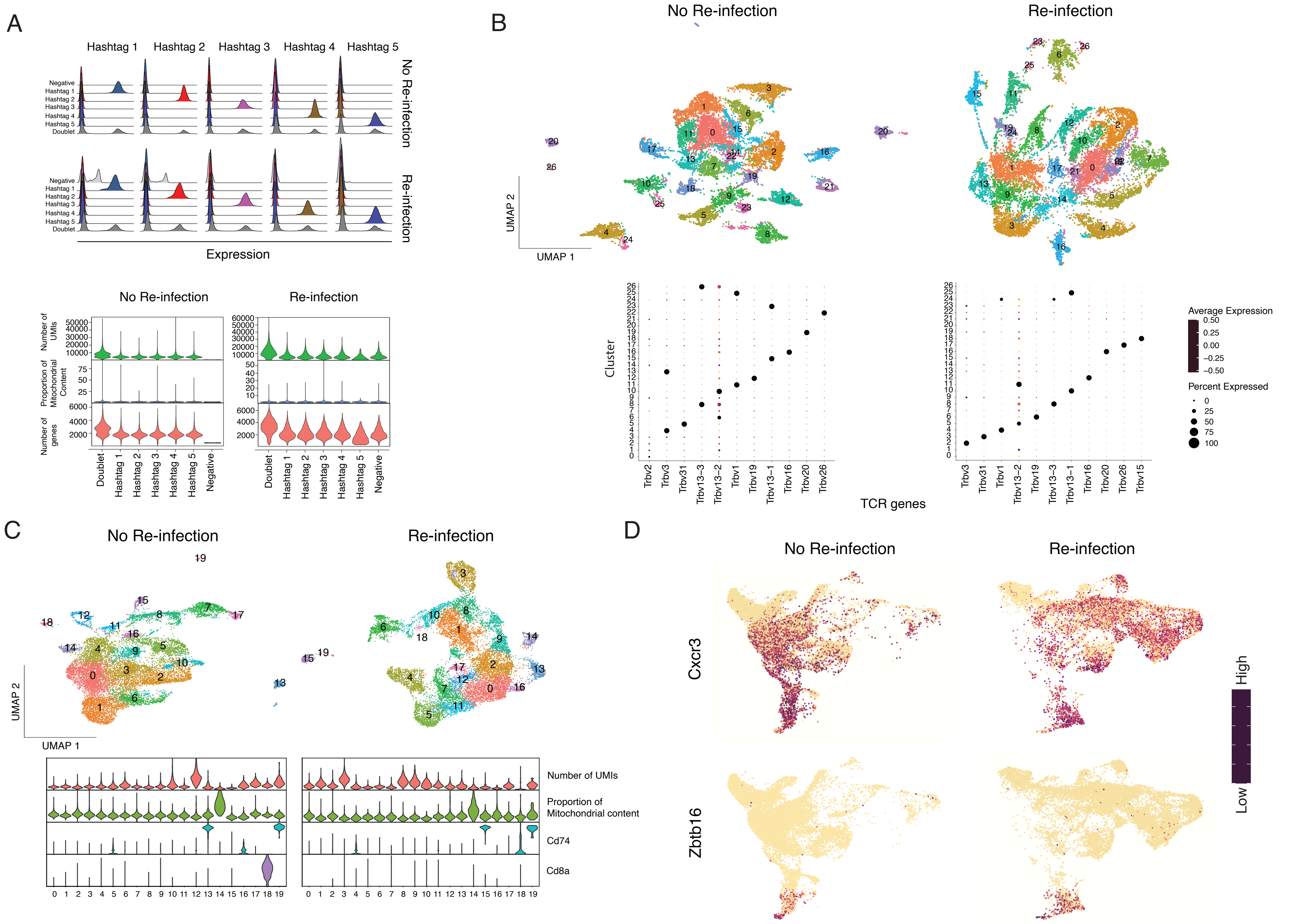
A
Hashtag 1
Hashtag 2
Hashtag 3
Hashtag 4
Hashtag 5
No Re-infection
Re-infection
Negative
Doublet
Expression
No Re-infection
Re-infection
Number of UMIs
Proportion of Mitochondrial Content
Number of genes
Doublet
Negative
B
No Re-infection
Re-infection
UMAP 2
UMAP 1
Cluster
Trbv2
Trbv3
Trbv31
Trbv13−3
Trbv13−2
Trbv1
Trbv19
Trbv13−1
Trbv16
Trbv20
Trbv26
Trbv15
TCR genes
Average Expression
Percent Expressed
C
No Re-infection
Re-infection
UMAP 2
UMAP 1
Number of UMIs
Proportion of Mitochondrial content
Cd74
Cd8a
D
No Re-infection
Re-infection
Cxcr3
Zbtb16
High
Low

Supplementary Figure 7: Clonal relationships of polyclonal CD4$^+$ T cells prior to and after re-infection
UMAP representation of CD11a^{hi}/CXCR3$^+$ polyclonal CD4$^+$ T cells (with 5% spike in of CD11a^{lo}/CXCR3$^-$ naïve cells) for 5 mice prior to re-infection, and 5 mice after re-infection. Cells sharing the same TCR chains are connected by edges and only families with four or more cells sharing the same TCR chains are shown on the plot. Each hashtag represents data from one mouse.

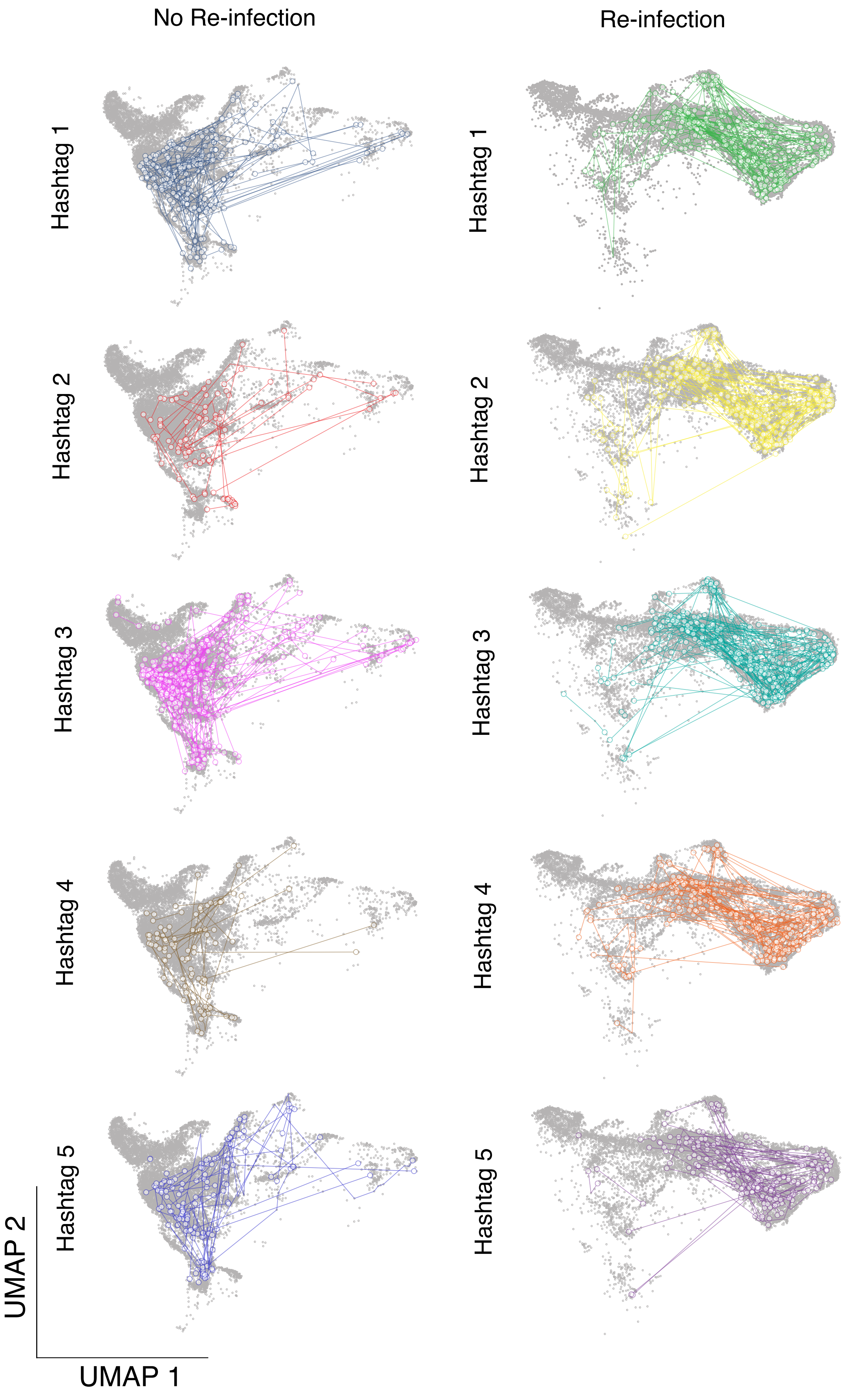
No Re-infection
Re-infection
Hashtag 1
Hashtag 2
Hashtag 3
Hashtag 4
Hashtag 5
UMAP 2
UMAP 1

Supplementary Figure 8: Antigen-experienced polyclonal CD4$^+$ T cells do not increase protein translation 24 hours after re-infection. (A) Representative FACS plots showing puromycin binding for paired naïve (CD11a^{lo} CXCR3^{lo}) naïve versus and activated polyclonal splenic CD4$^+$ T cells prior to and 24 hours after re-infection. Graph shows % puromycin$^+$ cells for individual mice (n=5 mice/group). Statistical analysis was performed using a non-parametric Mann-Whitney test (ns= not significant). Data representative of two independent experiments showing similar results. **(B)** Representative FACS plots of PD1 & CXCR5 expression in total (left panels) and puromycin$^+$ (right panels) activated, polyclonal CD4$^+$ T cells prior to and 24 hours after re-infection. Stacked bar graph showing the percentages of Tfh/Tcm-like, GC Tfh, and non-Tfh subsets. Data representative of two independent experiments showing similar results.

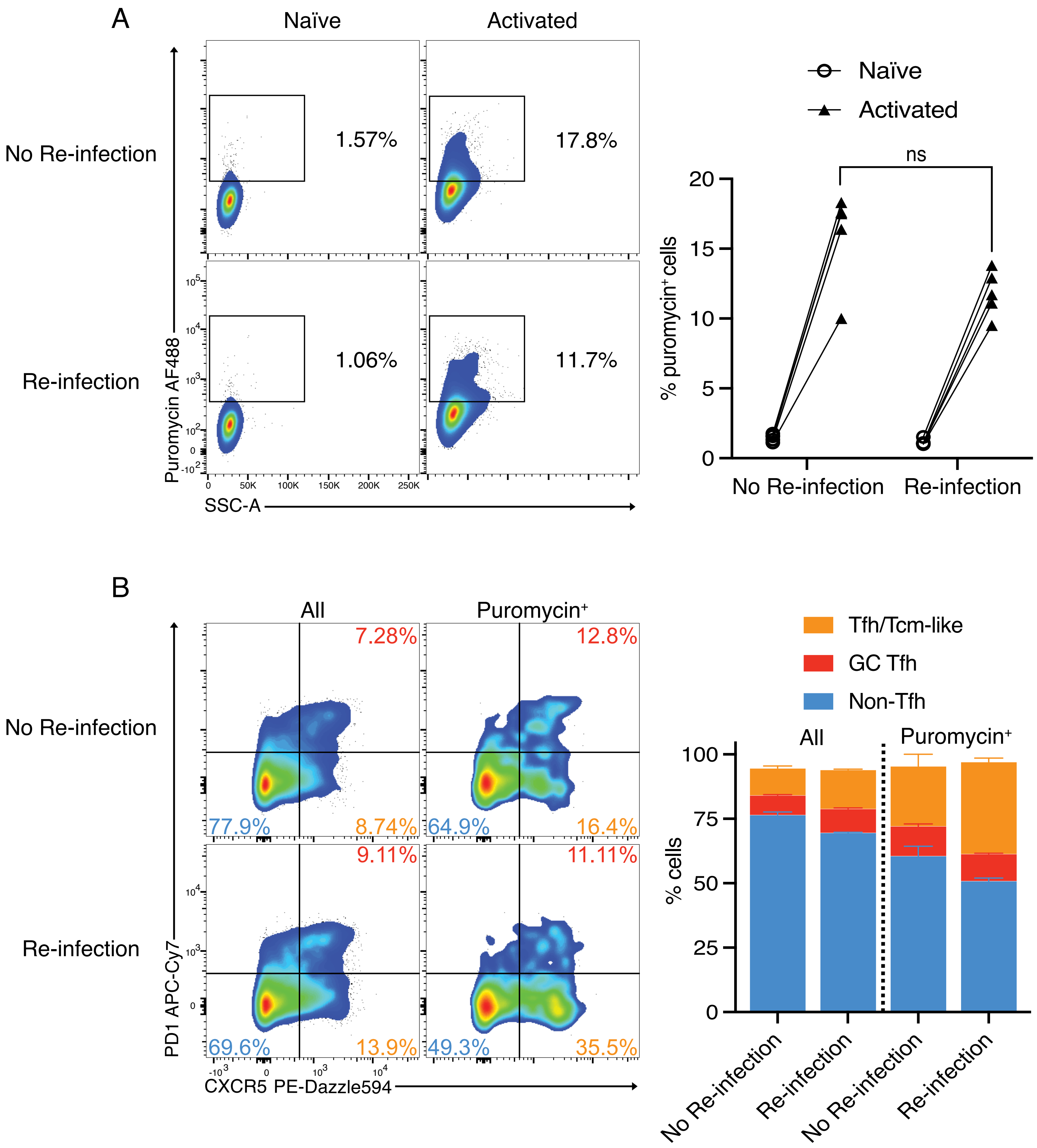
A
Naïve
Activated
No Re-infection
Re-infection
1.57%
17.8%
1.06%
11.7%
Puromycin AF488
SSC-A
0
50K
100K
150K
200K
250K
Naïve
Activated
ns
% puromycin⁺ cells
20
15
10
5
0
No Re-infection
Re-infection
B
All
Puromycin⁺
No Re-infection
Re-infection
7.28%
12.8%
77.9%
8.74%
64.9%
16.4%
9.11%
11.11%
69.6%
13.9%
49.3%
35.5%
PD1 APC-Cy7
CXCR5 PE-Dazzle594
Tfh/Tcm-like
GC Tfh
Non-Tfh
All
Puromycin⁺
% cells
100
75
50
25
0
No Re-infection
Re-infection
No Re-infection
Re-infection

Supplementary Figure 9: Quality control of scRNA-seq data for antigen- experienced and naïve PbTII cells prior to and 1 day after re-infection (A) Violin plots showing the distribution of PbTII cells for number of UMIs, proportion of mitochondrial content, and number of genes. **(B)** UMAP visualisation of *egfp* expression, denoting antigen- experienced PbTIIs.

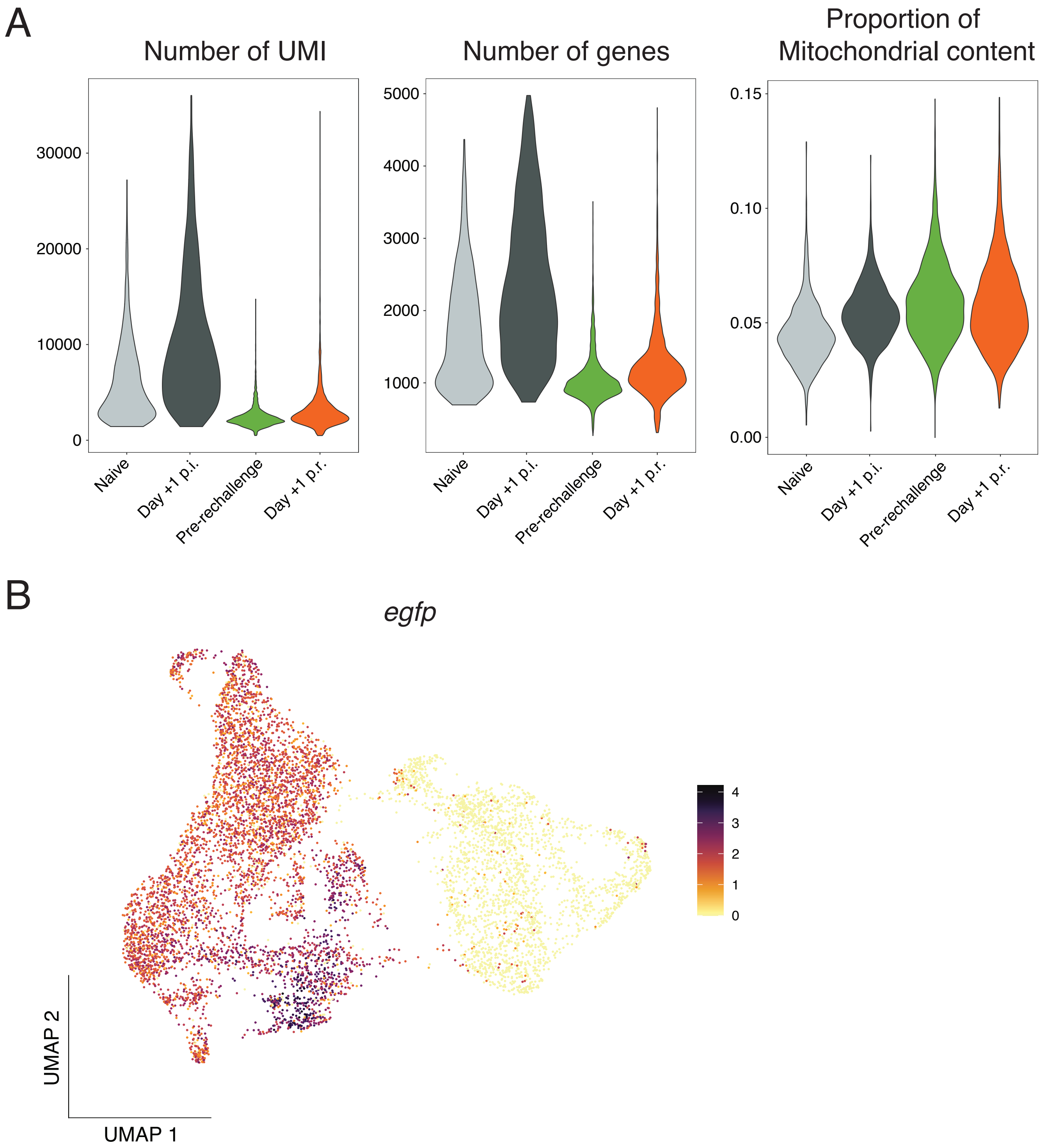
A
Number of UMI
0
10000
20000
30000
Naive
Day +1 p.i.
Pre-rechallenge
Day +1 p.r.
Number of genes
1000
2000
3000
4000
5000
Proportion of
Mitochondrial content
0.00
0.05
0.10
0.15
B
egfp
0
1
2
3
4
UMAP 2
UMAP 1

Supplementary Figure 10: Quality control of scRNA-seq data for PbTII cells prior to and 1, 2, and 3 days after re-infection. (A) Violin plots showing the distribution of PbTII cells for number of UMIs, number of genes, and G2M phase signature score for each cluster. **(B)** UMAP representation of PbTII cells before (Top) and after (Bottom) integration using scVI. Cells are coloured by clusters from unsupervised clustering (Leiden algorithm) (Left) and timepoint (Right). **(C)** Violin plots showing the distribution of PbTII cells for number of UMIs, proportion of mitochondrial content, number of genes, *and egfp* expression for each sample after removal of cluster 9 in **(A/B)**.

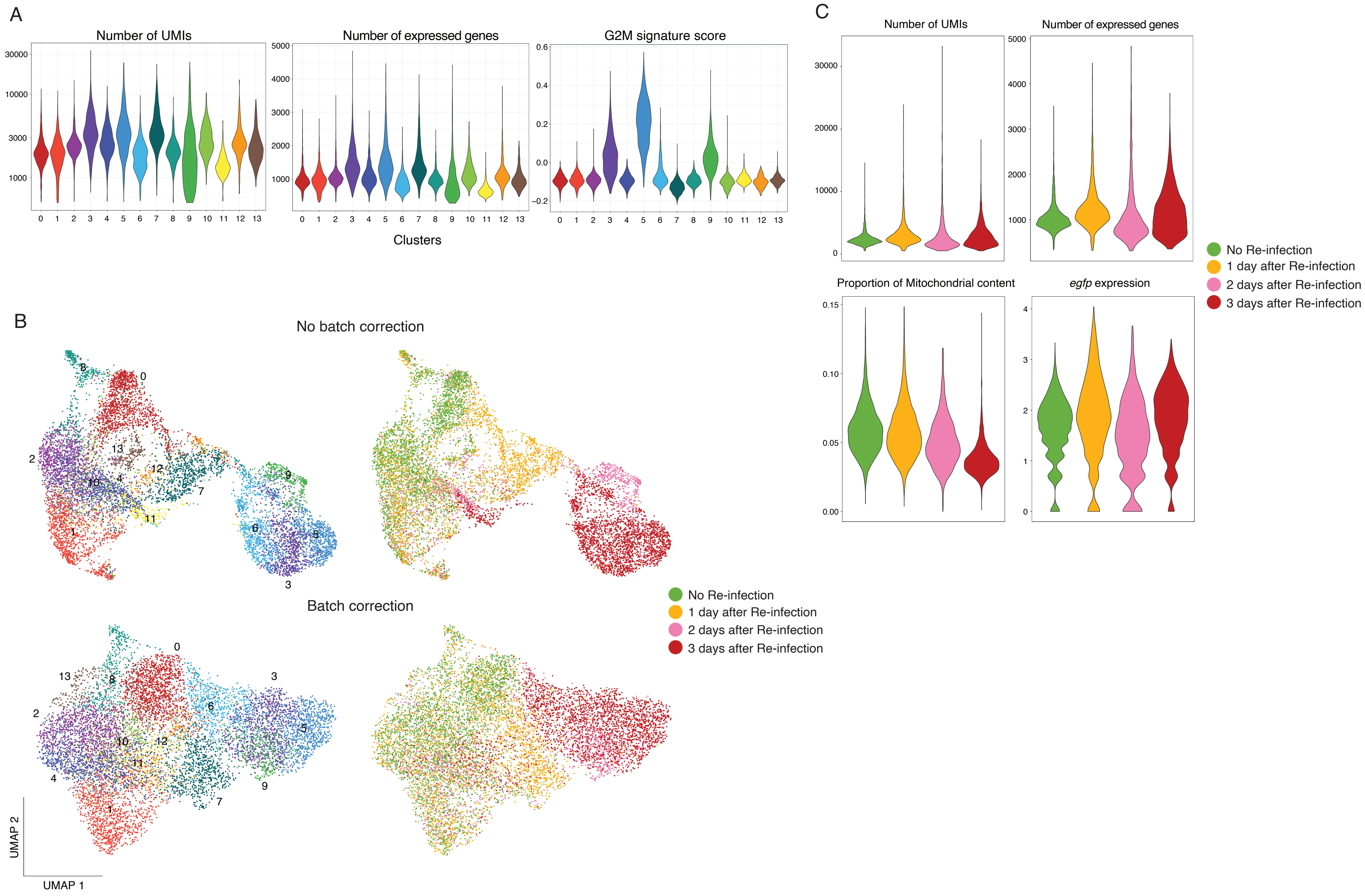
A
Number of UMIs
Number of expressed genes
G2M signature score
Clusters
B
No batch correction
Batch correction
UMAP 1
UMAP 2
No Re-infection
1 day after Re-infection
2 days after Re-infection
3 days after Re-infection
C
Number of UMIs
Number of expressed genes
Proportion of Mitochondrial content
egfp expression

Supplementary Figure 11: Analysis of scRNA-seq data of naïve and effector PbTII cells at day 7 post-infection, compared with day 28 post-infection (Soon *et al.* 2020). (A) UMAP representation of PbTII cells. Cells are coloured by sample of origin. **(B)** UMAP visualisation of PbTII cells expressing Th1 signature score. **(C)** (Left) UMAP representation of PbTII cells. Cells are coloured by clusters from unsupervised clustering (Louvain algorithm) and (Right) violin plot showing the distribution of PbTII cells for Th1 signature score for each cluster in **(B)**.

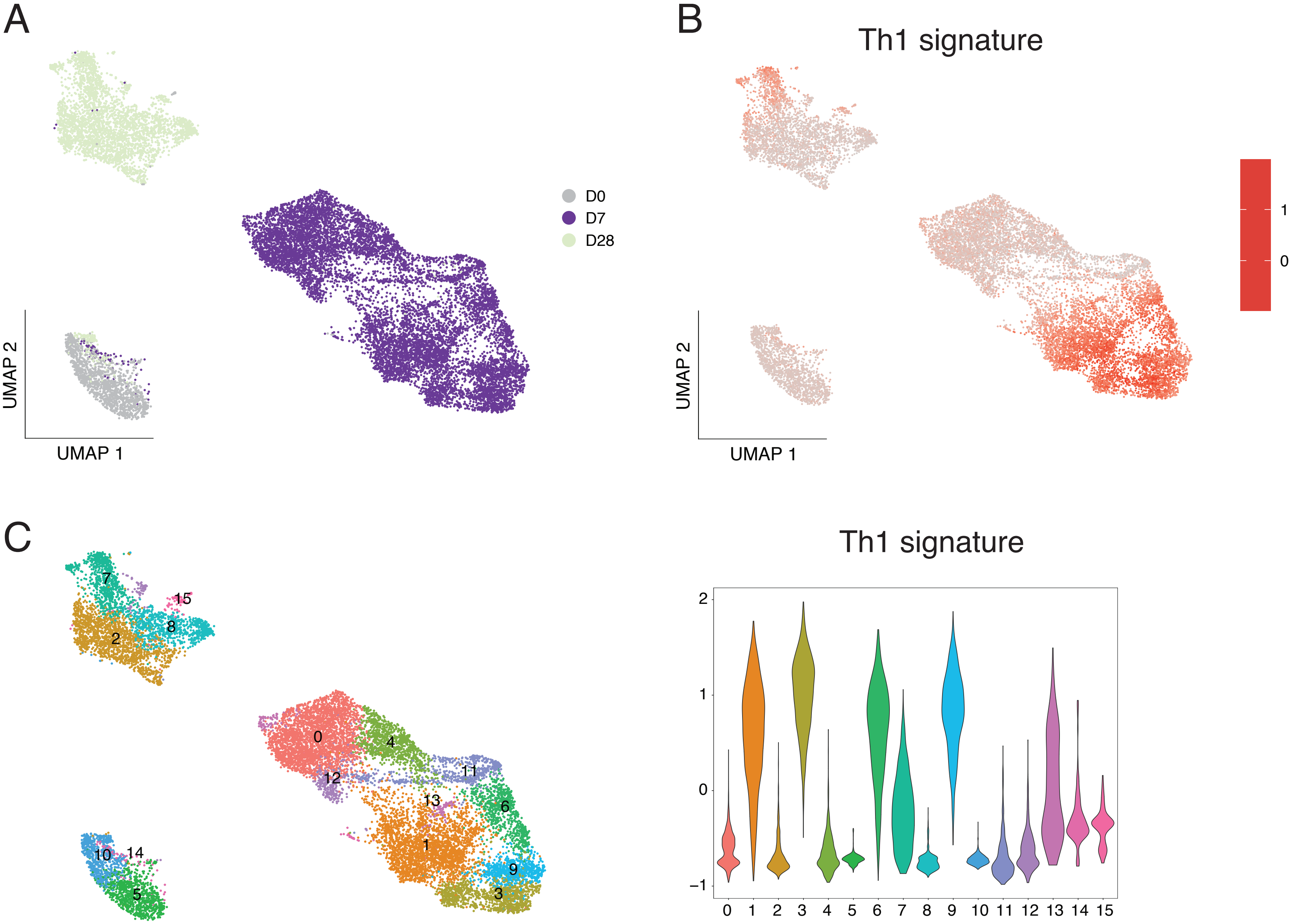
A
UMAP 2
UMAP 1
D0
D7
D28
B
Th1 signature
UMAP 2
UMAP 1
1
0
C
7
15
8
2
0
4
11
12
13
6
1
10
14
5
9
3
Th1 signature
2
1
0
−1
0
1
2
3
4
5
6
7
8
9
10
11
12
13
14
15

Supplementary Figure 12: Integration of scRNA-seq datasets of PbTII cells and polyclonal CD4$^+$ T cells prior to and 3 days after re-infection to identify cell clusters of similar phenotypes. (A) (Top) UMAP representation of PbTII cells and polyclonal CD4$^+$ T cells prior to data integration. Cells are coloured by clusters from unsupervised clustering. (Bottom) Combined and integrated UMAP representation generated using scVI. Cells are coloured by clusters from unsupervised clustering (Louvain algorithm) of PbTII cells (Left) and polyclonal CD4$^+$ T cells (Right). **(B)** Visualisation of Th1, Tcm, and Tfh signature scores on the integrated UMAP shown in (**A** – Bottom).

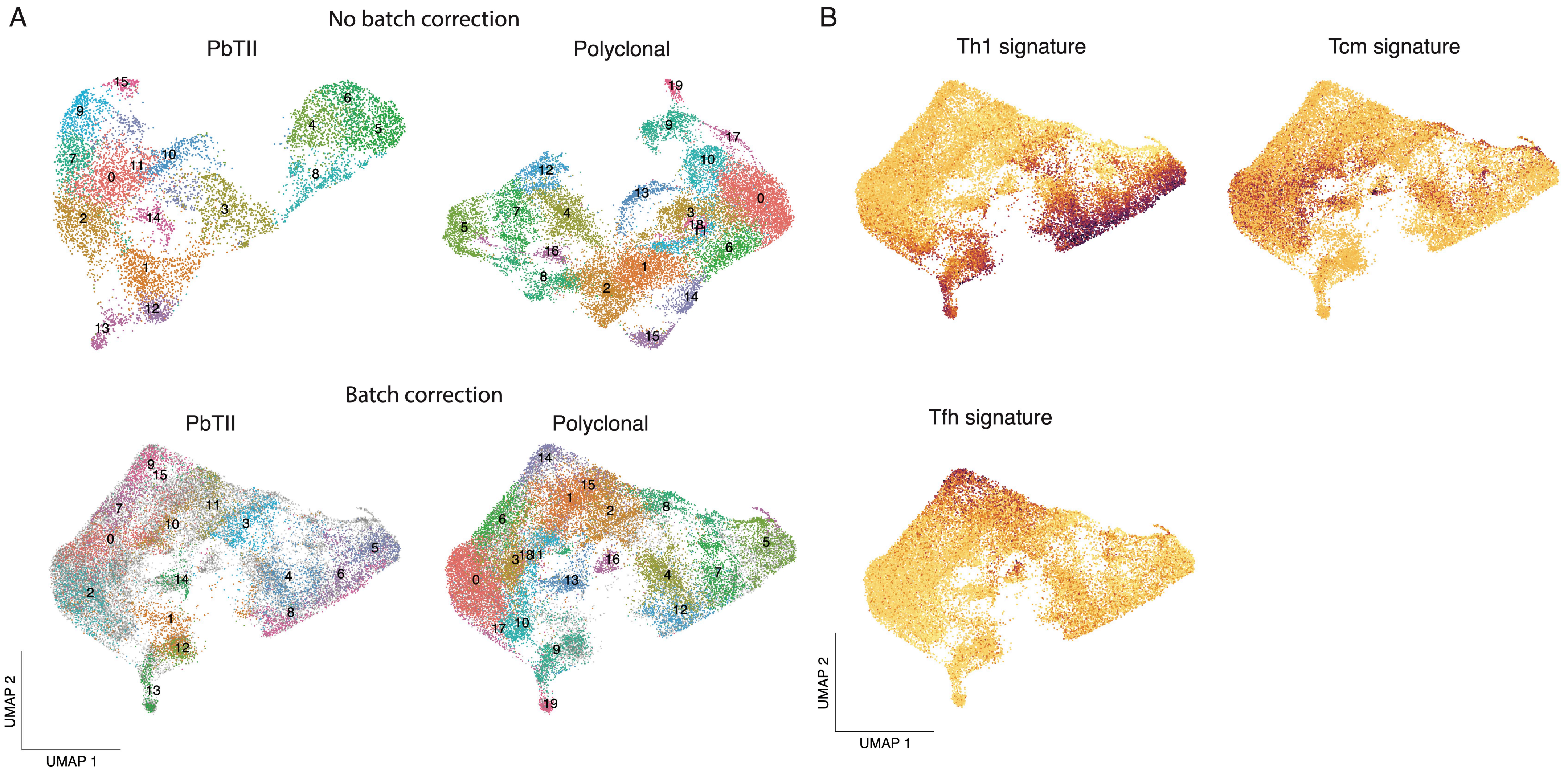
A
No batch correction
PbTII
Polyclonal
Batch correction
PbTII
Polyclonal
UMAP 2
UMAP 1
B
Th1 signature
Tcm signature
Tfh signature
UMAP 2
UMAP 1
