# Supplementary material for: CD4+ T cells display a spectrum of recall dynamics during re-infection with malaria parasites
Source: Nat Commun. 2024 Jun 28;15:5497. doi: 10.1038/s41467-024-49879-6 (PMC11214622; doi:10.1038/s41467-024-49879-6)
Supplement: Supplementary file 3 — Description of Additional Supplementary Files [file 41467_2024_49879_MOESM3_ESM.pdf]

Description of Additional Supplementary Files

Title: Supplementary Data 1

Description: List of differentially expressed genes between Th1 PbTII cells before and after re-infection.

Title: Supplementary Data 2

Description: List of differentially expressed genes between Tcm/Tfh PbTII cells before and after re-infection.

Title: Supplementary Data 3

Description: List of differentially expressed genes between GC Tfh PbTII cells before and after re-infection.

Title: Supplementary Data 4

Description: List of genes belonging to 9 individual dynamics.

Title: Supplementary Data 5

Description: List of genes upregulated in primary Th1 cells that are stably upregulated in memory and recall.

Title: Supplementary Data 6

Description: List of differentially expressed genes between Treg polyclonal cells before and after re-infection.

Title: Supplementary Data 7

Description: List of differentially expressed genes between Th1 polyclonal cells before and after re-infection.

Title: Supplementary Data 8

Description: List of differentially expressed genes between Tcm/Tfh PbTII cells before and after re-infection.

Title: Supplementary Data 9

Description: List of differentially expressed genes between GC Tfh polyclonal cells before and after re-infection.

Title: Supplementary Data 10

Description: List of monoclonal antibodies and hashtags employed
