# Supplementary material for: CD4+ T cells display a spectrum of recall dynamics during re-infection with malaria parasites
Source: Nat Commun. 2024 Jun 28;15:5497. doi: 10.1038/s41467-024-49879-6 (PMC11214622; doi:10.1038/s41467-024-49879-6)
Supplement: Supplementary file 5 — Reporting Summary [file 41467_2024_49879_MOESM5_ESM.pdf]

nature portfolio

Corresponding author(s): Ashraful Haque

Last updated by author(s): Mar 6, 2024

Reporting Summary

Nature Portfolio wishes to improve the reproducibility of the work that we publish. This form provides structure for consistency and transparency in reporting. For further information on Nature Portfolio policies, see our Editorial Policies and the Editorial Policy Checklist.

Statistics

For all statistical analyses, confirm that the following items are present in the figure legend, table legend, main text, or Methods section.

n/a	Confirmed	
☐	☒	The exact sample size (*n*) for each experimental group/condition, given as a discrete number and unit of measurement
☐	☒	A statement on whether measurements were taken from distinct samples or whether the same sample was measured repeatedly
☐	☒	The statistical test(s) used AND whether they are one- or two-sided<br>*Only common tests should be described solely by name; describe more complex techniques in the Methods section.*
☒	☐	A description of all covariates tested
☐	☒	A description of any assumptions or corrections, such as tests of normality and adjustment for multiple comparisons
☐	☒	A full description of the statistical parameters including central tendency (e.g. means) or other basic estimates (e.g. regression coefficient) AND variation (e.g. standard deviation) or associated estimates of uncertainty (e.g. confidence intervals)
☐	☒	For null hypothesis testing, the test statistic (e.g. *F*, *t*, *r*) with confidence intervals, effect sizes, degrees of freedom and *P* value noted<br>*Give P values as exact values whenever suitable.*
☒	☐	For Bayesian analysis, information on the choice of priors and Markov chain Monte Carlo settings
☒	☐	For hierarchical and complex designs, identification of the appropriate level for tests and full reporting of outcomes
☒	☐	Estimates of effect sizes (e.g. Cohen's *d*, Pearson's *r*), indicating how they were calculated

Our web collection on statistics for biologists contains articles on many of the points above.

Software and code

Policy information about availability of computer code

Data collection	Software used include: BD FACS DIVA software (version 8.0.1) was used for flow-sorting of cells and other relevant flow cytometry acquisition. Detailed parameters of methods are mentioned in the relevant sections in the Methods.
Data analysis	Software used includes:<br><br>Analysis of single cell RNAseq data was performed with 10x Genomics Cell Ranger (version 3.0.2 / version 6.1.1), Python (version 3), R (version 4.1.1), Seurat (version 4.1.0), scvi-tools (version 0.71.1), GPfates (https://github.com/Teichlab/GPfates), Harmony (version 0.1.0), Scanpy (version 1.9.1), spatialDE (version 1.1.3), GOrilla (http://cbl-gorilla.cs.technion.ac.il/) and REVIGO (version 1.8.1).<br><br>Analysis of slide-seqV2 data was performed with STAR (version 2.7.9a), R (version 3.5.1 / version 4.2.0), Seurat (version 3 / version 4), cell2location.<br><br>Analysis of all ATAC-seq data was performed with Python (version 3), R (version 4.1.1), BWA-MEM (https://github.com/lh3/bwa/blob/master/README.md), BEDTools (https://github.com/arq5x/bedtools2), MACS2 (version 2.2.6), UCSC Genome Browser, GenomicRanges (version 1.88.0), ChIPseeker (version 1.22.1), Picard (https://broadinstitute.github.io/picard/).<br><br>Cytometry data was acquired using FACSDiva (version 9.0). FlowJo (version 10.8.0) was used for analyzing flow cytometry data. Graph pad prism (version 9.1.2) and Adobe Illustrator (version 27.3.1) were used for visualization of the data.

For manuscripts utilizing custom algorithms or software that are central to the research but not yet described in published literature, software must be made available to editors and reviewers. We strongly encourage code deposition in a community repository (e.g. GitHub). See the Nature Portfolio guidelines for submitting code & software for further information.

Data

Policy information about availability of data

All manuscripts must include a data availability statement. This statement should provide the following information, where applicable:

- Accession codes, unique identifiers, or web links for publicly available datasets
- A description of any restrictions on data availability
- For clinical datasets or third party data, please ensure that the statement adheres to our policy

All raw sequencing data are available via the accession numbers: GSE233703, GSE233713 and GSE234253. Raw sequencing data from our previous publication used within our current study is available via the accession numbers: E-MTAB-9317, E-MTAB-9393, and E-MTAB-9402.

Research involving human participants, their data, or biological material

Policy information about studies with human participants or human data. See also policy information about sex, gender (identity/presentation), and sexual orientation and race, ethnicity and racism.

Reporting on sex and gender	N/A
Reporting on race, ethnicity, or other socially relevant groupings	N/A
Population characteristics	N/A
Recruitment	N/A
Ethics oversight	N/A

Note that full information on the approval of the study protocol must also be provided in the manuscript.

Field-specific reporting

Please select the one below that is the best fit for your research. If you are not sure, read the appropriate sections before making your selection.

[x] Life sciences [ ] Behavioural & social sciences [ ] Ecological, evolutionary & environmental sciences

For a reference copy of the document with all sections, see nature.com/documents/nr-reporting-summary-flat.pdf

Life sciences study design

All studies must disclose on these points even when the disclosure is negative.

Sample size	No statistical methods were used to predetermine sample size. We followed standards in the field to ensure statistical comparison was applicable.
Data exclusions	No mice were excluded based on pre-established criteria. No exclusion was applied to the uploaded raw data in GEO. For the final count matrix, we excluded cells based on pre-established criteria for single-cells: we excluded low quality samples and contaminating cells (i.e. - cells with low number of detected genes and high mitochondria content) - exclusion criteria for each case are detailed in the relevant Methods section.
Replication	All experimental repeats were successful.
Randomization	No active randomization was applied to experimental groups. The majority of sample/animal groups were defined by experimental treatment.
Blinding	None of our experiments were performed in a blinded fashion.

Reporting for specific materials, systems and methods

We require information from authors about some types of materials, experimental systems and methods used in many studies. Here, indicate whether each material, system or method listed is relevant to your study. If you are not sure if a list item applies to your research, read the appropriate section before selecting a response.

Materials & experimental systems

n/a	Involved in the study	
	x	Antibodies
x		Eukaryotic cell lines
x		Palaeontology and archaeology
	x	Animals and other organisms
x		Clinical data
x		Dual use research of concern
x		Plants

Methods

n/a	Involved in the study	
x		ChIP-seq
	x	Flow cytometry
x		MRI-based neuroimaging

Antibodies

Antibodies used

target / flurophore / clone / source / titration
CD4 / AF700 / RM4-5 / BioLegend / 1:200
CD4 / APC / RM4-5 / Biolegend / 1:200
CD4 / BV605 / RM4-5 / Biolegend / 1:200
CXCR3 / BV510 / CXCR3-173 / BioLegend / 1:200
CXCR5 / Biotin / 2G8 / BD / 1:50
CXCR5/PE-Dazzle594/L1387D/BioLegend/1:200
SAV / BV786 / / BioLegend / 1:200
CXCR6 / APC / SA051D1 / BioLegend / 1:200
CXCR6 / BV711 / SA051D1 / BioLegend / 1:200
CD11a / PerCP / Cy5.5 / M17/4 / BioLegend / 1:200
CD45.2 / AF700 / 104 / BD / 1:200
CD45.2 / PE-CF594 / 104 / BD / 1:200
CD45.2/BV421/104/BioLegend/1:200
TCRb / BUV737 / H57-5797 / BD / 1:200
TCRb / APC-Cy7 / H57-5797 / BD / 1:200
TCRVa2 / PE / B20.1 / BD / 1:200
TCRVb12 / PerCP-Cy5.5 / MR11-1 / eBioscience / 1:200
TCRVb12 / BV711 / MR11-1 / BD / 1:200
Ki67 / AF700 / SolA15 / eBioscience / 1:200
Ki67/AF647/SolA15/eBioscience/1:200
Ki67 / PE / SolA15 / eBioscience / 1:200
IFNg / BV421 / XMG1.2 / BioLegend / 1:200
IFNg / PE-CY7 / XMG1.2 / BioLegend / 1:200
Violet Proliferation Dye 450 / / BD / 1:1000

CD45/MHC-I / Hashtag_1 / M1/42; 30-F11; / BioLegend / 0.1-0.5ug/million cells
CD45/MHC-I / Hashtag_2 / M1/42; 30-F11; / BioLegend / 0.1-0.5ug/million cells
CD45/MHC-I / Hashtag_3 / M1/42; 30-F11; / BioLegend / 0.1-0.5ug/million cells
CD45/MHC-I / Hashtag_4 / M1/42; 30-F11; / BioLegend / 0.1-0.5ug/million cells
CD45/MHC-I / Hashtag_5 / M1/42; 30-F11; / BioLegend / 0.1-0.5ug/million cells

Validation

All antibody validation data (FACS plot) are found on manufacturer's website, in which relevant details are described above.

Animals and other research organisms

Policy information about studies involving animals; ARRIVE guidelines recommended for reporting animal research, and Sex and Gender in Research

Laboratory animals

All mice used were of the C57BL/6J background and were females aged 6-12 weeks old. Please refer to "Mice" section under Methods for further description.

Wild animals

N/A

Reporting on sex

All mice used were females.

Field-collected samples

N/A

Ethics oversight

The study was approved by the University of Melbourne Animal Ethics Committee (1915018) and QIMR Berghofer Animal Ethics Committee (A503-601M), in accordance to the guideline provided by the National Health and Medical Research Council of Australia.

Note that full information on the approval of the study protocol must also be provided in the manuscript.

Flow Cytometry

Plots

Confirm that:

- [x] The axis labels state the marker and fluorochrome used (e.g. CD4-FITC).
- [x] The axis scales are clearly visible. Include numbers along axes only for bottom left plot of group (a 'group' is an analysis of identical markers).
- [x] All plots are contour plots with outliers or pseudocolor plots.
- [x] A numerical value for number of cells or percentage (with statistics) is provided.

Methodology

Sample preparation	Spleens were collected and homogenised through a 70-100 µm cell strainer to create a single cell suspension and RBCs were lysed using RBC Lysing Buffer Hybri-Max™ (Sigma-Aldrich) or Pharm Lyse™ (BD). Splenocytes were assessed for viability using a LIVE/DEAD™ Fixable Aqua Dead Cell Stain Kit (Life Technologies) and Zombie Yellow viability dye (BioLegend), according to the manufacturer's protocol, unless otherwise specified. Prior to antibody staining, Fc receptors were blocked using antibodies against CD16 and CD32. Cells were incubated with titrated panels of monoclonal antibodies (Supplementary Table 10) diluted in PBS containing 1% of FCS and 2mM EDTA for 20 minutes at 4°C on dark. Intracellular staining was performed using the eBioscience™ Foxp3/Transcription Factor Staining Buffer Set or BD Cytofix/Cytoperm™ Fixation/Permeabilization Kit.
Instrument	Samples were acquired on a LSRII Fortessa analyser (BD Biosciences). Sorting was carried out on FACS Aria III (BD Biosciences).
Software	BD FACS DIVA software (version 9.0) was used for flow-sorting of cells and other relevant flow cytometry acquisition.
Cell population abundance	Transgenic CD4+ PbTII T cells were found to be in a range of 0.1% - 2.0% of all single, live CD4+ TCR+ cells from a population of enriched CD4+ T cells. This range varies due to the kinetics of expansion and contraction of CD4+ T cells during infection, characterised in Fig 1. These values are according to analysis of the FCS files recorded at the time of sorting or from FACS analysis only. Purity of sorted CD4+ PbTII cells was always determined post-sort to be >99.9% of the sorted population. Polyclonal CXCR3+/CD11a-high cell abundance varied from 19-30% on Day 30 post infection, and from 42-48% on day 3 post-reinfection. Purity of sorted polyclonal cells was determined post sort to be >98%.
Gating strategy	PbTII cells were identified as single, live (zombie aqua low), CD4+, TCR+ and GFP+ cells. Polyclonal cells were identified as single, live (zombie yellow low), CD4+, TCR-beta+, and CXCR3-/CD11a-low (naive), and CXCR3+/CD11a-high (experienced). All other markers of interest were gated against an isotype control where applicable or a naive infection control.

- [x] Tick this box to confirm that a figure exemplifying the gating strategy is provided in the Supplementary Information.
